# High production of valencene in *Saccharomyces cerevisiae* through metabolic engineering

**DOI:** 10.1186/s12934-019-1246-2

**Published:** 2019-11-07

**Authors:** Hefeng Chen, Chaoyi Zhu, Muzi Zhu, Jinghui Xiong, Hao Ma, Min Zhuo, Shuang Li

**Affiliations:** 1 School of Biology and Biological Engineering, South China University of Technology, Higher Education Mega Center, Guangzhou, 510006 China; 2 State Key Laboratory of Applied Microbiology Southern China, Guangdong Provincial Key Laboratory of Microbial Culture Collection and Application, Guangdong Institute of Microbiology, Guangzhou, 510070 China

**Keywords:** Valencene, Synthetic biology, Metabolic engineering, *Saccharomyces cerevisiae*, Recyclable plasmid, Expression cassette

## Abstract

**Background:**

The biological synthesis of high value compounds in industry through metabolically engineered microorganism factories has received increasing attention in recent years. Valencene is a high value ingredient in the flavor and fragrance industry, but the low concentration in nature and high cost of extraction limits its application. *Saccharomyces cerevisiae*, generally recognized as safe, is one of the most commonly used gene expression hosts. Construction of *S. cerevisiae* cell factory to achieve high production of valencene will be attractive.

**Results:**

Valencene was successfully biosynthesized after introducing valencene synthase into *S. cerevisiae* BJ5464. A significant increase in valencene yield was observed after down-regulation or knock-out of squalene synthesis and other inhibiting factors (such as *erg9*, *rox1*) in mevalonate (MVA) pathway using a recyclable CRISPR/Cas9 system constructed in this study through the introduction of Cre/loxP. To increase the supplement of the precursor farnesyl pyrophosphate (FPP), all the genes of FPP upstream in MVA pathway were overexpressed in yeast genome. Furthermore, valencene expression cassettes containing different promoters and terminators were compared, and P_HXT7_-VS-T_TPI1_ was found to have excellent performance in valencene production. Finally, after fed-batch fermentation in 3 L bioreactor, valencene production titer reached 539.3 mg/L with about 160-fold improvement compared to the initial titer, which is the highest reported valencene yield.

**Conclusions:**

This study achieved high production of valencene in *S. cerevisiae* through metabolic engineering and optimization of expression cassette, providing good example of microbial overproduction of valuable chemical products. The construction of recyclable plasmid was useful for multiple gene editing as well.

## Background

Valencene, a natural sesquiterpene, possesses good biological activity and is found in various citrus species, such as in the essential oil of Valencia orange [1]. When used in flavoring and fragrances, valencene imparts a woody citrus characteristic and can be economically used as an additive in food and drinks [2]. Besides, valencene can be further derivatized into many economically useful sesquiterpenes, such as its oxidation product nootkatone which can inhibit proliferation of cancer cells. Valencene has broad application prospects in food, cosmetics and pharmaceutical industries [3]. However, it is not economically feasible to extract valencene in large-scale from natural sources due to its low concentration in citrus fruits and high cost of purification [4]. Constructing microbial cell factories to carry out the production of valencene is more attractive on account of the short growth cycle and low cost.

Previous studies have achieved high valencene yield through expression of valencene synthase gene (*CnVS*) in genetically engineered *Rhodobacter sphaeroides* strains, including expression of mevalonate operon from *Paracoccus zeaxanthinifaciens* [5]. Frohwitter et al. constructed recombinant *Corynebacterium glutamicum* containing valencene synthase from *Nootka cypress* and genes *erg20* and *ispA* were overexpressed, resulting in valencene yield of 2.41 mg/L [6]. In previous studies, *Saccharomyces cerevisiae* has been considered an ideal host for metabolic engineering of valencene production due to its favorable physiological properties, and optimized fermentations produced maximal levels of 20 mg/L valencene [4, 7, 8]. However, the yield of valencene in *S. cerevisiae* is still unable to meet the industrial demand, which indicates the need for achieving higher yields through metabolic engineering.

All terpenes are biosynthesized through mevalonate pathway (MVA) in yeast, originating from acetyl-CoA, and the intermediate product farnesyl diphosphate (FPP) is the direct precursor of valencene. FPP is a key metabolic point in MVA pathway toward the metabolism branches, such as the synthesis of squalene with squalene synthase encoded by *erg9* [9] and synthesis of GGPP with geranylgeranyl diphosphate synthase encoded by *bts1* [10], leading to synthesis of isoprenes [11]. It is reported that *rox1* is a transcriptional factor inhibiting the expression of hypoxia-induced genes in MVA pathway and ergosterol biosynthesis [12, 13]. Moreover, some distantly located genetic loci might have potential interactions with some terpenoid pathway, such as *ypl062w* and *yjl064w* [7, 14]. Recent research pointed out that the *ypl062w* functioned as an important promoter for *ald6* whose expression level was negatively correlated with terpenoid productivity [15]. Apart from pathway engineering, the selection of appropriate expression cassettes including promoter and terminator is also important for the optimization of valencene production [16]. This is because the transcription level is determined by the promoter strength, and also because promoters behave differently under different growth conditions. Stephanopoulos and coworkers systematically tested promoters of different strengths and plasmid copy-numbers, and were able to maximize taxadiene production to approximately 1 g/L with minimal accumulation of any toxic intermediate [17–19]. Thus, it is attractive to test the yield of valencene using different expression cassettes.

**Fig. 1:**
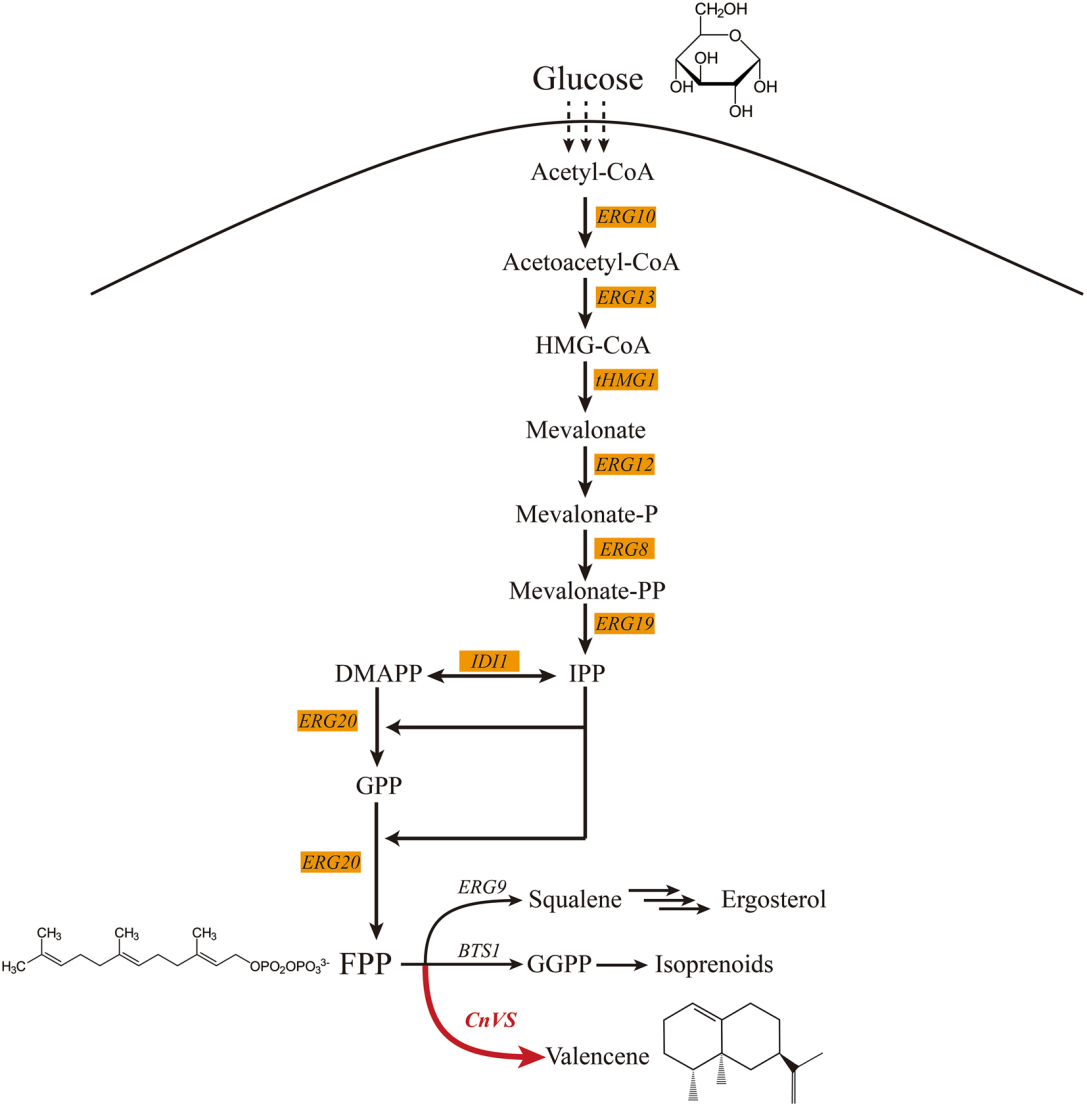
The schematic of engineered mevalonate (MVA) pathway and relevant downstream pathways in *S. cerevisiae*. The heterologous valencene synthetic pathway is highlighted in red. *ERG10*, acetyl-CoA C-acetyltransferase; *ERG13*, hydroxymethylglutaryl-CoA synthase; *ERG12*, mevalonate kinase; *tHMG1*, truncated 3-hydroxy-3-methylglutaryl-CoA reductase; *ERG8*, phosphomevalonate kinase; *ERG19*, diphosphomevalonate decarboxylase; *IDI1*, isopentenyl-diphosphate delta-isomerase; *ERG20*, farnesyl diphosphate synthase/dimethylallyltranstransferase; *ERG9*, squalene synthase; *BTS1*, geranylgeranyl diphosphate synthase; *CnVS*, valencene synthase from *Callitropsis nootkatensis*

In this study, *S. cerevisiae* was selected as the host to construct cell factory and achieve the overproduction of valencene. A series of metabolic engineering strategies were performed in MVA pathway (Fig. 1). Firstly, a recyclable CRISPR/Cas9 system was constructed to achieve multiple genome editing to accumulate the FPP pool toward the synthesis of valencene. Secondly, to enhance the metabolic flux to precursor FPP, the key genes of FPP synthetic pathway in MVA pathway were overexpressed in yeast genome. Thirdly, valencene synthase expression cassette was optimized through constructing a promoter-terminator library. Using the above combinatorial engineering strategies, this work offers a good reference to increase the heterologous expression performance of valuable compounds in *S. cerevisiae* through metabolic engineering.

## Results and discussion

### Construction of recyclable plasmid and application in CRISPR/Cas9 system

CRISPR/Cas9 system, the essential components of which are gRNA expression plasmid and Cas9 protein, is the most widely used genome editing technology for prokaryotes and eukaryotes due to its efficient genome editing ability [20–22]. However, when using CRISPR/Cas9 system to perform multiple genetic modifications, such as gene knock-out, knock-in or tagging, there are bottlenecks because of the limited selection markers on each gRNA expression plasmid. To solve this problem, some marker recycling systems are used currently, such as FLP/FRT system [23, 24], Cre/loxP system [25], counter-selection system [26, 27] and I-SceI [28].

In this study, a recyclable plasmid mediated by Cre/loxP system was constructed. First, under the control of galactose-inducible promoter GAL1, the Cre recombinase gene was introduced into plasmid p426, and subsequently, two loxP direct repeats were placed flanking 2μ replication origin of plasmid p426, resulting in the recyclable plasmid P426-CL (Additional file 1: Fig S1). When the strain containing P426-CL was cultivated in galactose medium, expression of Cre recombinase was induced. This mediated the excision of the 2μ replication origin on P426-CL, leading to the lack of replication capacity. The plasmid P426-CL was lost within cells in the passage and the selection marker of *URA* could be repeatedly used, which meant genome editing could proceed again in strains that had been edited by recyclable gRNA expression plasmid. As the existence of selection marker *URA* is important for the efficiency of subsequent gene editing, it is necessary to verify the plasmid loss efficiency. The efficiency results are shown in Table 1. It can be seen that, after 36 h culturing in galactose medium, the ratio of plasmid-free cells was observed to be nearly 100% (Table 1), which was higher than that obtained using Cre/loxP in yeast *Pichia* (65%) [29]. Thus, the engineered yeast strain could be applied to a new round of genome editing using the gRNA vector (based on P426-CL) targeting another gene of interest (knock-in or knock-out). Ryan et al. [30] reported a scarless and marker-free genome editing method based on CRISPR-Cas9 in *S. cerevisiae*. However, the plasmid that coexpresses the Cas9 endonuclease and guide RNA (sgRNA) expression cassette will still be remained in the host cell, and the selection marker on the plasmid could not be recycled. In the study of Stovicek et al. [31], researchers focused on the development of the genetic manipulation on unrelated prototrophic polyploidy *S. cerevisiae* strains with the efficiency ranging between 65% and 78%. Although it is claimed that marker-free recombinants were isolated through natural loss of plasmid, the loss efficiency was not stable and satisfactory. The high loss rate of P426-CL under galactose induction gave us a guarantee on the recyclable CRISPR/Cas9 system.

**Table 1:** The loss efficiency of recycling gRNA expression plasmid P426-CL

Dilution factor (D)	Colony number				Loss efficiency (%)
	Plate 1	Plate 2	Plate 3	Average	
10^3^	12	15	47	25	99.98
10^4^	1	3	2	2	99.98
10^5^	0	0	1	0	100

The plasmid loss efficiency was calculated based on following equation ${\text{Plasmid loss efficiency}} = \frac{{A_{1} - D \times \left( {A_{2} \div 0.2} \right)}}{{A_{1} }}$

A_1_ = The density of yeast solution, CFU/mL, A_2_ = The colony number in the solid plate, D = The dilution factor. Each dilution factor was done in triplicate

**Fig. 2:**
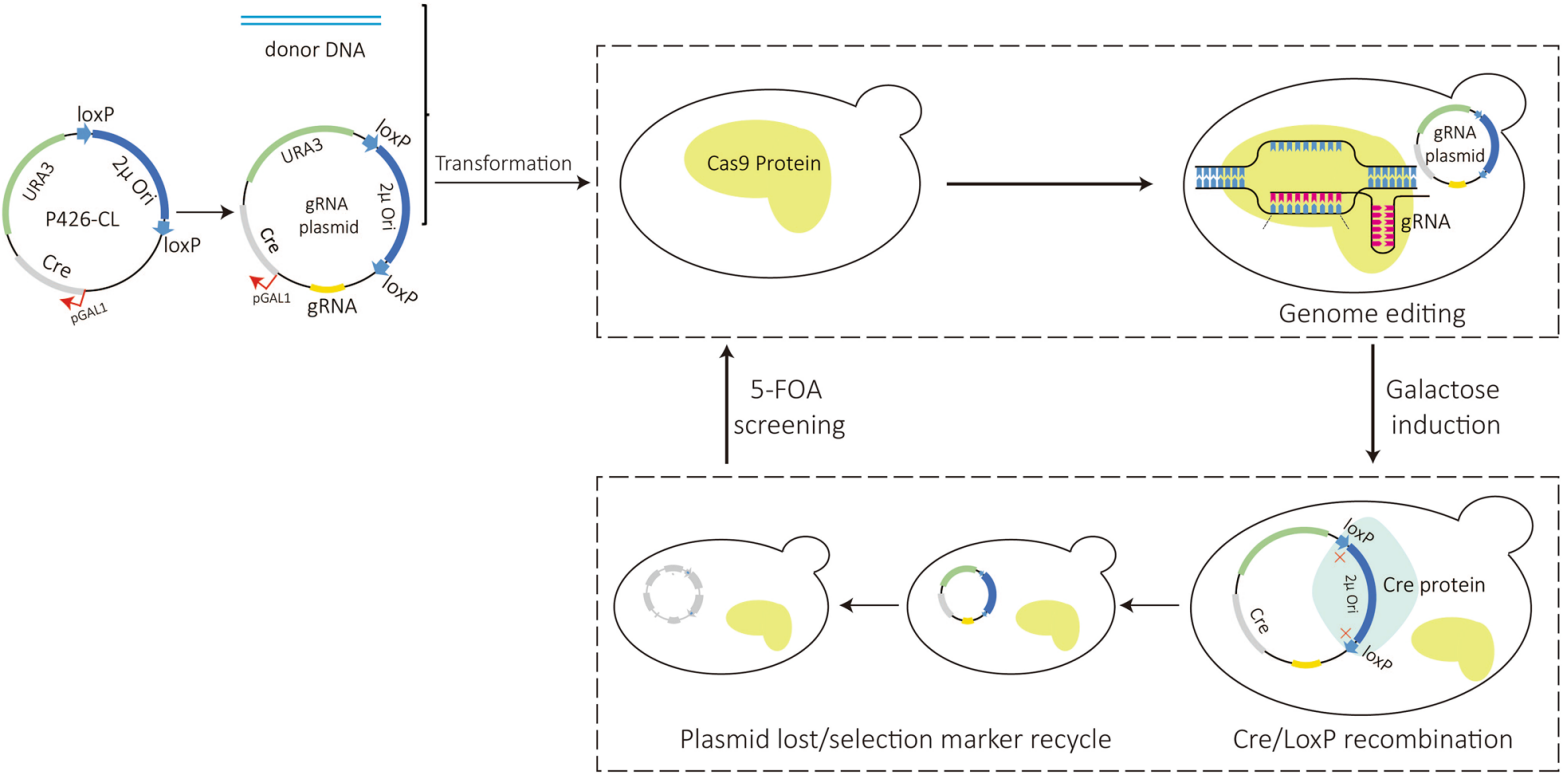
The schematic of gene editing using CRISPR/Cas9 system mediated by recyclable plasmid P426-CL. (i) single gRNA expression plasmid was constructed based on P426-CL. The recyclable gRNA expression plasmid was transformed into cells containing Cas9 protein to perform gene editing with homologous donor DNA. (ii) after successful gene editing, genetically modified strain was cultivated in galactose medium to induce expression of Cre recombinase. The 2μ replication origin between two loxP loci in gRNA expression plasmid was excised by Cre recombinase. The gRNA expression plasmid was lost in cells along with passage. (iii) the yeast culture in galactose medium was plated onto SD-glucose/5-FOA agar plate. Cells without gRNA expression plasmid grew out and were selected for preparation of competent cell. The new gRNA expression plasmid could be transformed into the competent cell for next round of gene editing

Based on recyclable plasmid P426-CL, five gRNA expression plasmids (Additional file 1: Table S1) were constructed to target different gene loci (*bts1*, *rox1*, *erg9*, *ypl062w* and *yjl064w*) (Additional file 1: Table S2) that may influence valencene production in FPP upstream. Then, 31 mutant combinations were obtained through repeating use of these five gRNA expression plasmids and recycling of selection marker (Fig. 2). To evaluate the efficiency of mutant combinations, 10 colonies of each genome editing strain were selected for genomic PCR and sequencing. The results (Additional file 1: Fig S2) showed that the mutant efficiency of almost all mutant combinations was 100%, which showed a higher genome editing success rate than that reported by Jakočiūnas et al. [11], especially in multiple sites editing. Only five single gRNA expression plasmids were required to achieve 31 mutant combinations instead of constructing many gRNA expression plasmids for each mutant, which may save a lot of work [11]. More practically, with the help of recyclable CRISPR/Cas9 system, new genetic modifications can be made directly based on the optimized strains instead of repeating the previous strategies first.

### Regulation of FPP downstream for valencene production

**Fig. 3:**
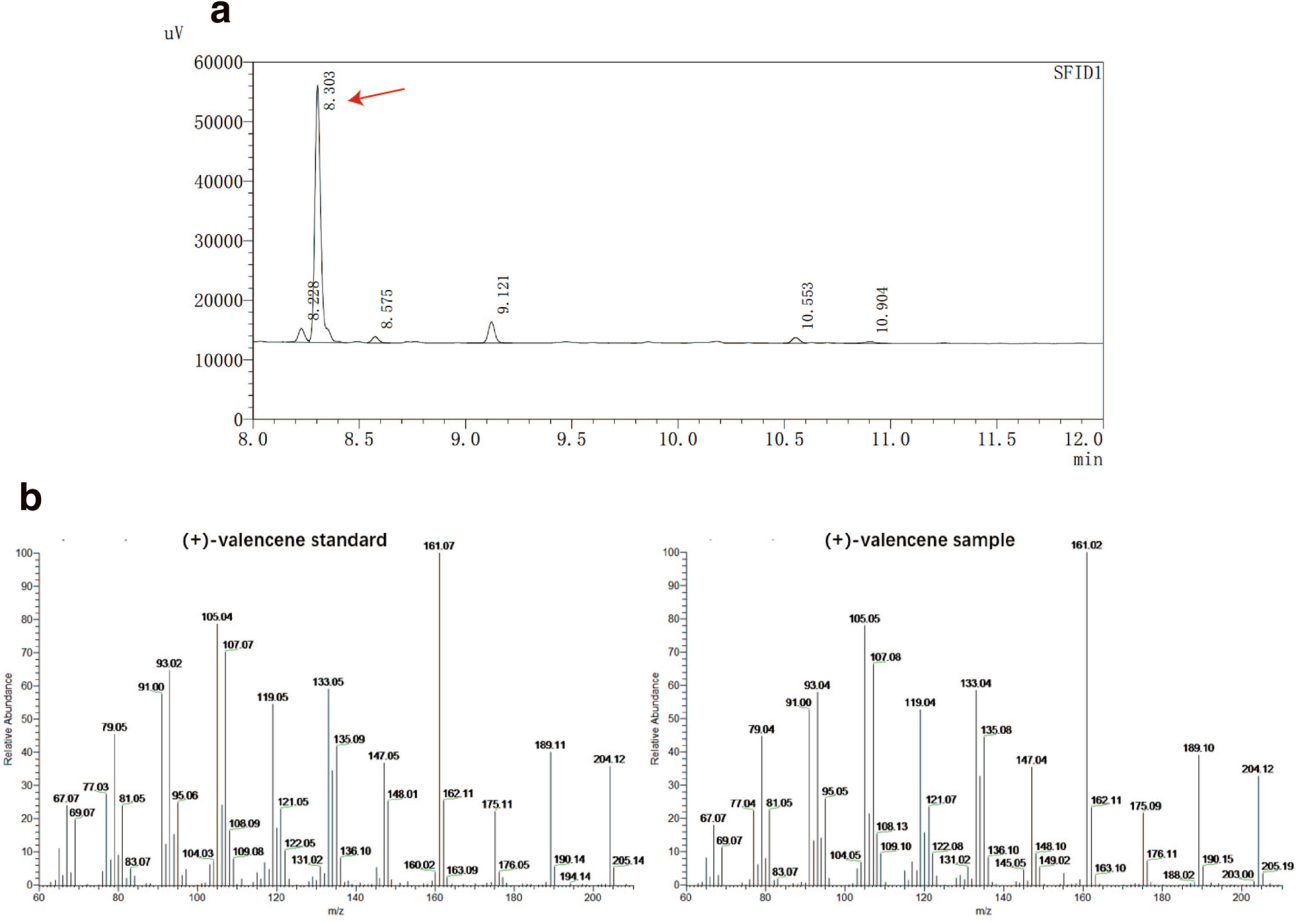
The GC profile of valencene produced by yeast strain. **a** Detected by GC-FID, the peak of valencene is shown by red arrow. **b** Detected by GC–MS, valencene is shown by the 204 m/z ion in the chromatographic trace

**Fig. 4:**
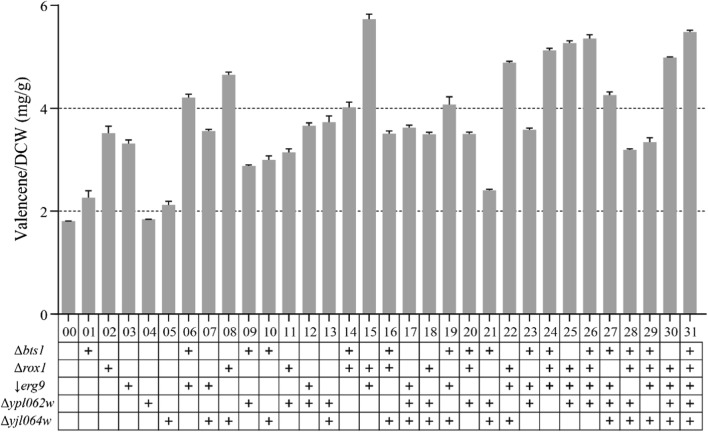
The valencene yields of 31 genome editing strains compared to wild type strain. Wild type and 31 recombinant strains were cultivated in shake flask at 30 °C for 48 h. The valencene yield was determined through GC–MS. Strains from BJM-00 to BJM-31 were abbreviated from 00 to 31

After successful multiple genome editing of five targeted gene loci that would influence valencene synthesis, the valencene yields of wild-type strain and 31 mutant strains (Additional file 1: Table S3) were examined through fermentation in shake flasks. Valencene synthase from *Callitropsis nootkatensis* (*CnVS*) was introduced into the recombination strain. The detection of valencene was performed by GC-FID and GC–MS (Fig. 3). From the fermentation results (Fig. 4), it was found that all mutant strains obtained higher yields of valencene compared to wild type and the highest strain gave 5.7 mg/g DCW, which was a 3.2-fold increase. As shown in previous study [11], the single knock-out of *rox1* gene improved valencene yield more than other single gene mutant. Moreover, all the 16 mutant strains with knock-out of *rox1* showed significant enhancement on valencene yield. Besides, *erg9* repression through the deletion of 45-bp (coordinates are − 220 to − 175) in the range of upstream activating *cis*-element (UAS) in *erg9* promoter resulted in obvious increase in yield of valencene in both single and multiple mutants. For the ergosterol synthesis charged by *erg9* is necessary to *S. cerevisiae* survival, researchers often tried to downregulate the *erg9* expression instead of blocking the pathway thoroughly. It has been demonstrated that the alteration of UAS could obviously reduce the *ERG9* expression [32]. When the deletion of *rox1* and alteration of UAS in *erg9* promoter were combined, the increase of valencene production was more significant, indicating the effect of two mutations were synergistic.

Among these 31 strains, higher multiple genome editing generally showed higher increase in yield of valencene than lower multiple genome editing. For instance, the quadruple mutant *rox1*;*erg9*;*ypl062w*;*yjl064w* (2.8-fold) showed higher increase in yield compared to triple mutant *erg9*;*ypl062w*;*yjl064w* (twofold). However, some combinatorial mutants did not show this pattern. For example, the quadruple mutant *bts1*;*rox1*;*erg9*;*yjl064w* (1.9-fold) showed much lower increase in yield than triple mutant *rox1*;*erg9*;*yjl064w* (2.7-fold), and *bts1*;*rox1*;*ypl062w*;*yjl064w* (1.8-fold) showed lower increase than triple mutant *rox1*;*erg9*;*ypl062w* (2.9-fold). This showed that the interaction between the five genes was not always positive under different genetic backgrounds.

The other three single mutants, *bts1*, *ypl062w*, and *yjl064w,* did not show significant increase in yield of valencene, even though knock-out of *bts1* was supposed to restrict metabolic flow to isoprenoid and promote valencene synthesis. The squalene synthase encoded by *ERG9* has a *K*_m_ value of 2.5 μM for FPP and a *k*_cat_ number of 0.53/s [33], while geranylgeranyl diphosphate (GGPP) synthase encoded by *BTS1* has a *K*_m_ value of 3.2 μM for FPP and a *k*_cat_ number of 0.025/s [34]. The higher *K*_m_ value for FPP and low turnover number of GGPP synthase indicate its low capacity. This may be the reason why the knock-out of *bts1* was less effective than down-regulation of *erg9*.

These findings suggested that the gene alteration of *erg9*, *rox1* and *bts1* was beneficial to valencene production. While, knock-out of *ypl062w* and *yjl064w* showed weak effect on valencene synthesis in *S. cerevisiae*.

### Overexpression of FPP synthetic pathway in MVA pathway

FPP is derived from the MVA pathway as precursor of valencene synthesis. However, the native FPP synthetic pathway has a low synthetic capacity that limits the production of valencene [35]. The overexpression of endogenous truncated 3-hydroxy-3-methylglutaryl coenzyme A reductase (tHMG1), a key enzyme in MVA pathway, has been shown to increase the biosynthesis of FPP [36]. However, few studies focus on the overexpression of other key genes in FPP synthetic pathway.

**Fig. 5:**
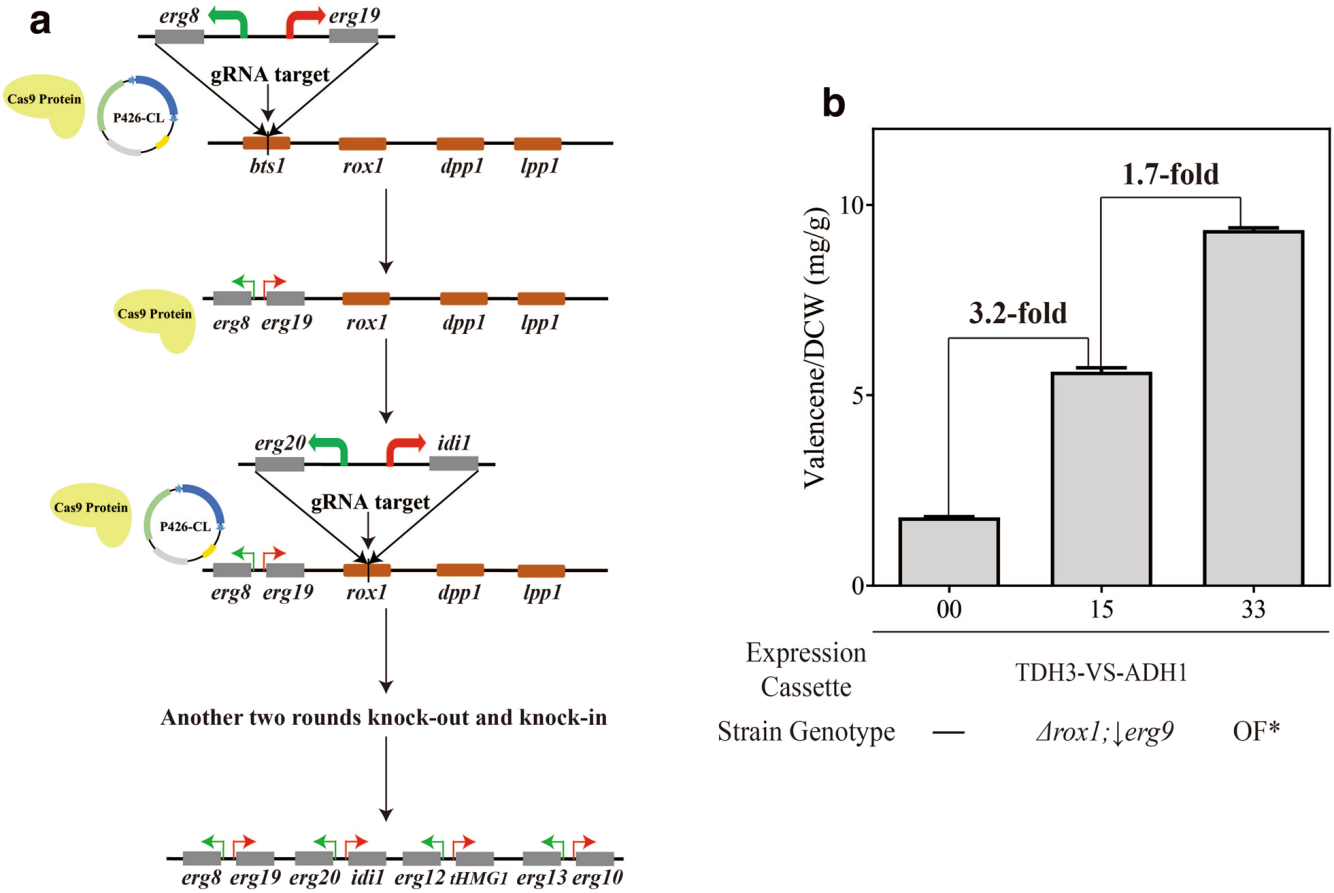
Overexpression of FPP synthetic pathway and the yield determination of overexpression strain. **a** 8 genes in FPP synthetic pathway were overexpressed in yeast genome using recyclable CRISPR/Cas9 system. Four gene loci (*bts1*, *rox1*, *dpp1*, *lpp1*) were chosen for integration of FPP synthetic genes. **b** The fermentation result in shake flask detected by GC–MS. OF* stands for overexpression of FPP synthetic pathway

To enhance FPP synthesis, every gene in FPP synthetic pathway (*erg10*, *erg13, tHMG1*, *erg12*, *erg8*, *erg19*, *erg20* and *idi1*) was integrated into the genome of BJM-03, in which the *ERG9* expression was down-regulated. Aforementioned results indicated that alteration in *ypl062w* and *yjl064w* showed no obvious benefit on valencene production. While the *dpp1* and *lpp1* were reported to be responsible for the hydrolysis of FPP and their knock-out could enhance FPP supplementation [37]. Based on this information, the integration sites for were finally selected as *bts1*, *rox1*, *dpp1* and *lpp1*. Through the selection marker recyclable plasmid P426-CL, BJM-33 were obtained after four rounds of gene knock-out/integration (Fig. 5a).

The recombinant strains were cultivated in shake flask and valencene yield was determined by GC-FID. The result showed that strain BJM-33 increased valencene production by 5.2-fold to 9.4 mg/g DCW compared with wild-type strain BJM-00 (Fig. 5b). However, compared to strain BJM-15 which possessed the best valencene production in 31 mutant strains (Fig. 4), the yield only increased by 1.7-fold, which was not in accordance with expectation. In a previous study, geraniol production was increased by 1.4-fold through overexpression of only *tHMG1* [38]. In other works, augmentation of the native yeast genes in MVA pathway increased nerolidol production by 3.5-fold [39]. The capacity of valencene synthase was suspected to be the limiting factor of increasing valencene production rather than the precursor FPP pool. The amount of FPP may have exceeded the conversion capacity of valencene synthase. So increasing valencene synthase expression may be necessary for high valencene yield.

### Screening of promoter-terminator pairs for valencene production

**Fig. 6:**
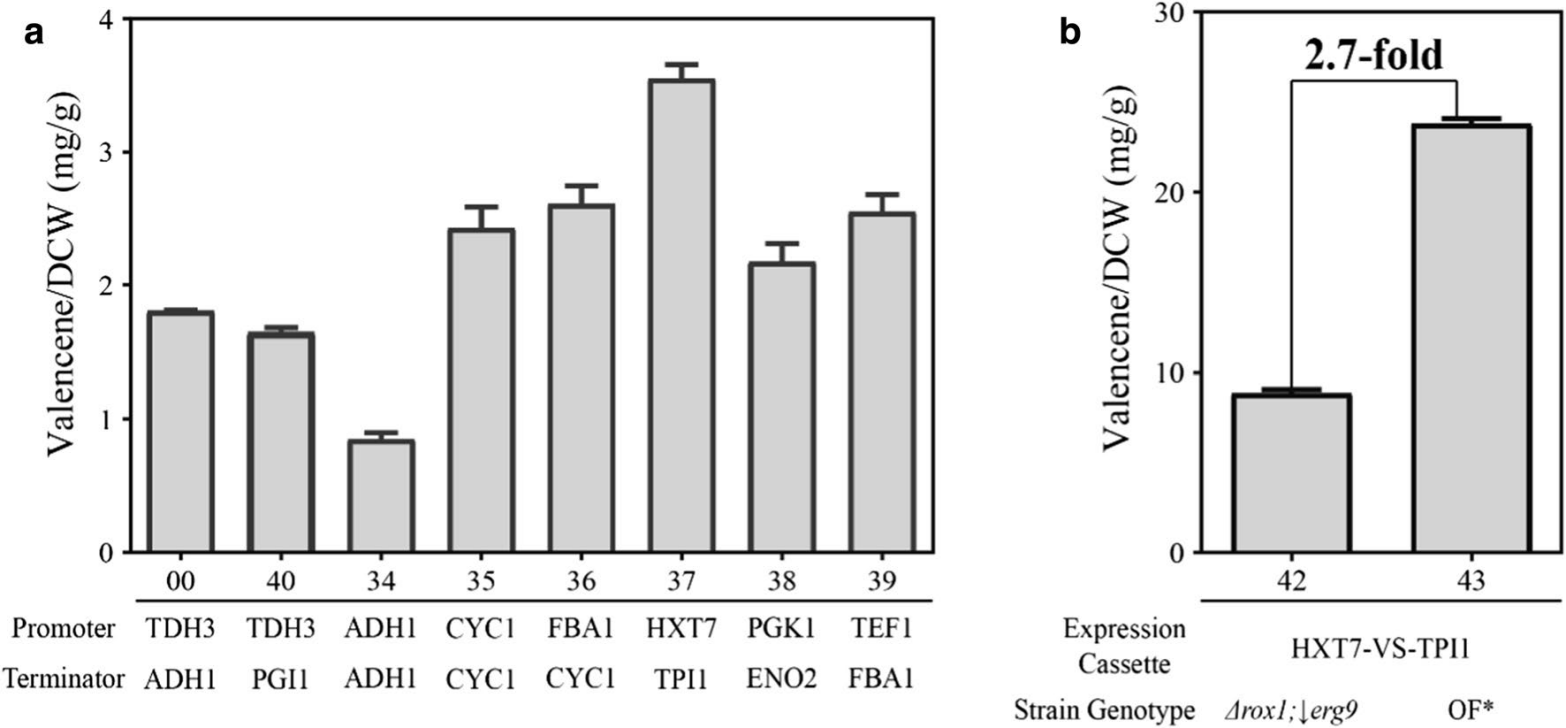
The screening of *CnVS* expression cassette and influence on valencene yield. **a** Several promoters and terminators from yeast genome were chosen to construct different *CnVS* expression cassettes. Recombinant BJ5464 strains harboring different *CnVS* expression cassettes were cultivated in shake flask compared to initial strains. **b** The *CnVS* expression cassette P_HXT7_-VS-T_TPI1_ with the best valencene yield was introduced into the strains BJM 15 and strain BJM 33 respectively, resulting in strains BJM 42 and BJM 43. OF* stands for overexpression of FPP synthetic pathway. The prefix BJM is omitted from the strain names for brevity

The above work focused on the regulation of MVA pathway, which showed some increase in yield of valencene. However, choosing a suitable *CnVS* expression cassette containing different promoters and terminators would influence the production of target compounds as well. In this study, a promoter-terminator library consisting of 7 combinations was constructed, and *CnVS* gene was introduced using restriction enzymes (labeled as P_ADH1_-VS-T_ADH1_, P_CYC1_-VS-T_CYC1_, P_FBA1_-VS-T_CYC1_, P_HXT7_-VS-T_TPI1_, P_PGK1_-VS-T_ENO2_, P_TDH3_-VS-T_PGI1_, P_TEF1_-VS-T_FBA1_). The recombination strains containing different *CnVS* expression cassettes were cultivated in shake flask to evaluate the change in valencene yield compared to strain BJM-00 (P_TDH3_-VS-T_ADH1_). According to the results shown in Fig. 6a, the strengths of the 8 promoters could be ranked in the following order: P_HXT7_ > P_FBA1_ > P_TEF1_ > P_CYC1_ > P_PGK1_ > P_TDH3_ > P_ADH1_.

The above result is contrary to the result reported by Partow et al. that P_TEF1_ showed strongest activity [40]. Moreover, Jie Sun et al. [16] compared 14 constitutive promoters from *S. cerevisiae* and found that P_TEF1_ was the strongest and P_PGI1_ was the weakest. The inconsistent result implied that the activity of a promoter may not be the same in different expression cassettes or in different product’s heterologous expression. Interestingly, P_TDH3_-VS-T_ADH1_ and P_TDH3_-VS-T_PGI1_, consisting of the same promoter but different terminators, also showed different valencene yields (1.8 mg/g DCW and 1.7 mg/g DCW), which indicated that both promoter and terminator could influence valencene synthase transcription level. As for promoter HXT7, Partow et al. [40] found that the activity of P_HXT7_ was low when glucose concentration was high and it increased along with consumption of glucose. Previous work also showed that P_HXT7_ was a high-affinity hexose transporter which was highly expressed at low glucose concentration (< 4.4 mM) [41, 42].

In order to combine the advantages of FPP supply improvement and expression cassette optimization, the best performing plasmid YM10 containing P_HXT7_-VS-T_TPI1_, was transformed into the strain BJM-42 and BJM-43, generating BJM-44 and BJM-45, respectively. After 48 h cultivation, valencene production was dramatically increased as 22.7 mg/g DCW in BJM-45, which is increased by 2.7 fold compared with BJM-44 (Fig. 6b). The result showed that overexpression of FPP upstream in MVA pathway had a large influence on valencene yield with an optimized expression cassette. The high performance of BJM-45 also indicated the effectiveness of combinatorial engineering strategies in valencene synthesis, which might be meaningful for biotechnological production of other valuable chemicals. The increase in yield caused by optimization of *CnVS* expression cassette also implied that the activity or expression of *CnVS* may be the main limiting factor. Modification of the enzyme and searching for new valencene synthase would be useful strategies for further improvement.

### The fed-batch fermentation of valencene overproduction

**Fig. 7:**
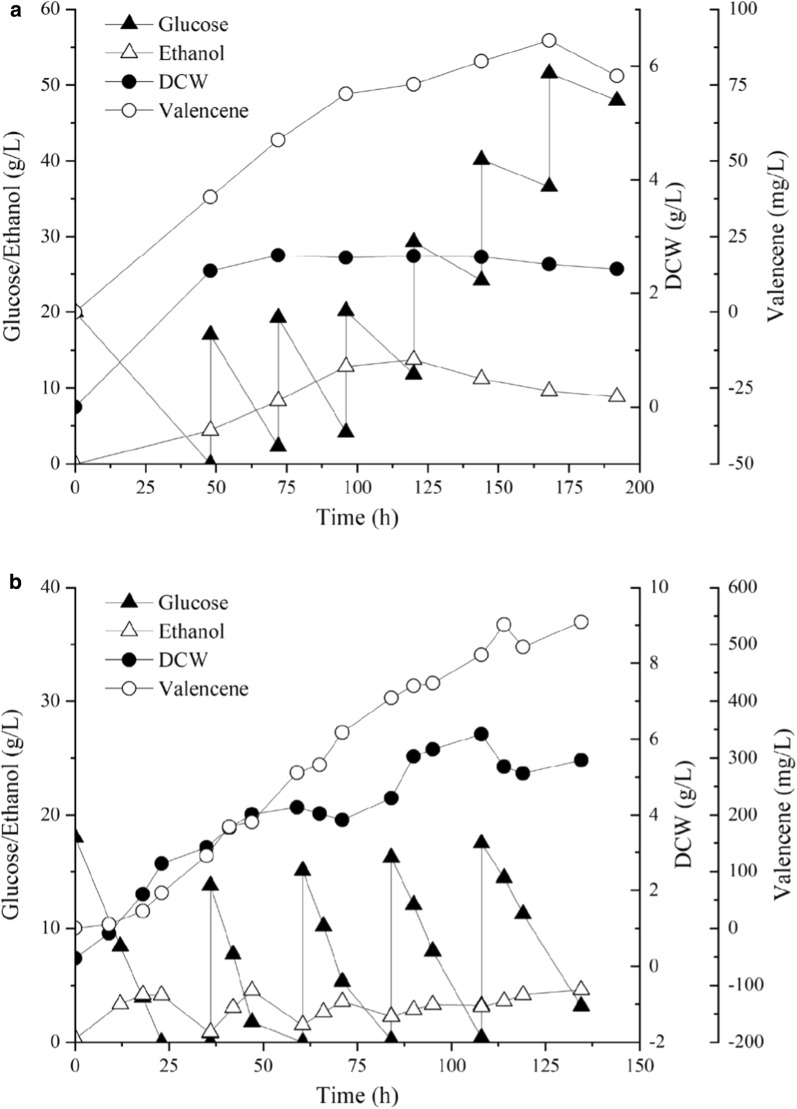
Time course profiles of glucose (solid triangle), ethanol (open triangle), dry cell weight (DCW, open circle), and valencene yield (solid circle) of strain *S. cerevisiae* BJM-45 using 20 g/L glucose as initial carbon source at 30 °C. **a** Fed-batch fermentation in 50 mL shake flask for 192 h with 6 times supplement of glucose. **b** Fed-batch fermentation in 3 L bioreactor for 135 h with 5 times supplement of glucose

To evaluate the production performance of the engineered strain BJM-45, fed-batch fermentation was performed with SD-glucose/ΔLeu medium in shake flask. After fermentation for 192 h with 6 times supplement of glucose, the highest valencene yield of 89.7 mg/L was obtained (Fig. 7a). The result showed that the strain basically stopped growing after 48 h and the residual sugar concentration in the medium kept increasing. The low dissolved oxygen in shake flask may cause this problem and restrict the high production of valencene.

Fed-batch fermentation was performed at a 1 L scale medium in 3 L bioreactor to determine the best performance of optimized strain BJM-45. After 136 h with 4 times supplement of glucose, the highest valencene titer of 539.3 mg/L was obtained, which was sixfold higher than that of fed-batch fermentation in shake flask (Fig. 7b). It was also much higher than the yields reported previously in engineered *S. cerevisiae* strains [4, 43] or in other valencene producers, such as *Corynebacterium glutamicum* [44] and *Schizophyllum commune* [45] (Table 2).

**Table 2:** Comparison of valencene yield in different engineered host strain

Host strain	Genetic engineering	(+)-valencene (mg/L)	References
*R. sphaeroides*	–	57.5	[5]
	Mevalonate operon from *P. zeaxanthinifaciens*	352	
*S. cerevisiae* WAT11	–	1.36	
*S. cerevisiae* WAT11	*ura3*, *leu2*, *trp1*, *his3*, *erg9*, *sue* mutation	20	[4]
*C. glutamicum*	Overexpression of *ERG20*	0.15	[6]
	Overexpression of *ispA*	0.22	
	Overexpression of *ispA*	2.41	
*P. pastoris*^a^	–	51	[52]
	Overexpression of *tHMG1*, *ADH1*	166	
*S. commune*	*thn* mutation	16.6	[45]
*S. cerevisiae*	–	0.18	[53]
	Overexpression of *tHMG* Copy addition of *CsTPS1* Expression of *mtCsTPS1*, *mtFDPS*	1.5	
*S. cerevisiae* BJ5464	Overexpression of *erg10*, *erg13*, *tHMG1*, *erg12*, *erg8*, *erg19*, *erg20* and *idi1* *Δrox1*, down-regulated *erg9*, *Δbts1*, *Δdpp1*, *Δlpp1*	539.3	This study

^a^Except for valencene production, β-nootkatol and nootkatone were also synthesized through valencene biotransformation. The total terpenoids titer is 418 mg/L

The course profiles in bioreactor (Fig. 7b) showed that glucose was almost entirely consumed and the biomass increased by twofold compared to shake flask. However, the cell density was still relatively low in fed-batch experiment and far below high-density fermentation (30 g/L DCW), as the highest CDW only reached 6 g/L throughout the fermentation. Additionally, the yeast cells at the later period of fermentation were collected for microscopic observation and it was found that most of the cells were dead (Additional file 1: Fig S3). In future, increasing the cell density in fed-batch fermentation and improving the cytoactivity of yeast cells at the later period of fermentation would be useful measures. Intracellular and extracellular metabolomics analysis would be an efficient way to find suitable biomarkers for batch media or for optimization of feed solution. It is expected that valencene production by the engineered strain in this study could be further improved by continuous efforts in both metabolic engineering and fermentation optimization.

## Conclusions

In this study, the high production of high value compound valencene was achieved through metabolic engineering using modified genome editing technology. Valencene synthase from *Callitropsis nootkatensis* (*CnVS*) was introduced into yeast, achieving valencene biosynthesis. A recyclable plasmid mediated by Cre/loxP system was constructed and applied in CRISPR/Cas9 system to perform multiple genetic modifications. The FPP pool toward valencene synthesis was enhanced through knock-out or down-regulate of gene loci of pathway branch or inhibiting factor. The valencene yield was increased to some extent through these strategies, but the metabolic flux in MVA pathway still needs further study and precise regulation. The effect of overexpression of FPP synthetic pathway into yeast genome was estimated through shake flask fermentation., and some increase was obtained. Moreover, it was found that the expression cassette P_HXT7_-VS-T_TPI1_ showed significant influence on valencene yield. To achieve further optimization of expression cassette, more regulation work is required in the future. The best valencene titer (539.3 mg/L) were achieved in fed-batch of 3 L bioreactors, which was highest production reported so far. Specific valencene yield was improved by approximately 160-fold compared to the initial strain. This study provides a good reference for microbial overproduction of valuable chemical products through combinatorial pathway engineering and optimization of expression cassette.

## Materials and methods

### Chemicals and media

All the heterologous genes and primers used in this study were obtained from Sangon (Shanghai, China). PrimerSTAR Max Premix used for fragment cloning was purchased from TaKaRa (Dalian, China). DNA purification and plasmid isolation kits were purchased from Tiangen (Beijing, China). T4 DNA ligase and restriction endonuclease were purchased from Thermo fermentas (MD, USA). The *S.c* EasyComp Transformation kit used for yeast transformation was provided by Invitrogen (CA, USA). Standards of valencene were obtained from Sigma (Sigma-Aldrich, MO, USA). KOD FX, High Success-rate DNA polymerase, was used in colony PCR to screen positive clones and was purchased from TOYOBO (Japan).

*Escherichia coli* DH5α were grown in Luria–Bertani (LB) medium containing 10 g/L tryptone, 10 g/L NaCl and 5 g/L yeast extract. Agar was added into the medium with concentration of 20 g/L if solid plate was needed. The yeast strains were grown in Synthetic Dropout (SD) medium containing 6.2 g/L Do Supplement (Clontech, USA), 67 g/L Yeast Nitrogen Base without amino acids (YNB) with 20 g/L glucose or 20 g/L galactose as carbon source (denoted as SD-glucose and SD-galactose, respectively), minus the auxotrophic amino acids complemented by recombinant plasmids. SD-glucose medium supplemented with 1 g/L 5-fluoroorotic acid (5-FOA) was used for selecting yeast colonies which showed loss of Orotidine 5′-phosphate decarboxylase (ODCase, encoded by *URA3*) activity.

### Construction of plasmids and strains

#### Construction of recyclable plasmid

The *cre* gene was PCR amplified with primer pair Cre-F and Cre-R (Additional file 1: Table S4) from plasmid pUC57 synthesized by Sangon (Shanghai). The Cre recombinase expression cassette was constructed under the control of the inducible Gal1 promoter in the plasmid YEplac181 between *Sma*I site and *Bam*HI site. The expression fragment P_GAL1_-Cre-T_CYC1_ was amplified by PCR using primer pair of GAL1-F and CYC1-R and cloned into plasmid p426-P_SNR52_-gRNA.CAN1.Y-T_SUP4_ (shortened as P426, Addgene (USA)) using ClonExpress II One Step Cloning Kit (Vazyme, China), resulting in the plasmid p426-Cre. To introduce the loxP direct repeats into both sides of 2μ replication origin of plasmid, the p426-Cre was linearized using primer pair PB-F and Ori-R. Here, both primers contained part of loxP direct repeat sequence as homologous arms. Then, the linearized fragment was self-ligated using ClonExpress II One Step Cloning Kit and one loxP direct repeat was introduced into 3′ side of 2μ replication origin of the plasmid. Another loxP direct repeat sequence was inserted using primer pair of Ori-F and PB-R in the same way, and plasmid P426-CL was obtained.

#### Valencene expression plasmid construction

The valencene synthase gene from *Callitropsis nootkatensis* (*CnVS*) was synthesized by Sangon (Shanghai) with codon optimization and inserted into plasmid YEplac181 (*LEU2* selection marker) under the control of TDH3 promoter between *Sma*I and *Bam*HI restriction sites, yielding plasmid pYM06. The plasmid was transformed into yeast strain BJ5464 using standard protocols.

#### Knock-out and knock-down strains construction

To improve the valencene production, 5 gene loci that could influence metabolic flux toward valencene synthesis were chosen for metabolic engineering. Among these 5 gene loci, *rox1* [12, 13], *bts1* [43, 46], *ypl062w* and *yjl064w* [47] were knocked out, and *erg9* [48, 49] was down-regulated. Then, 31 combinations of these 5 gene loci from single mutants to quintuple mutants were designed.

To obtain these 31 mutant constructions, 5 gRNA expression plasmids, P426-bts1, P426-rox1, P426-erg9, P426-ypl062w, and P426-yjl064w were constructed based on recyclable plasmid P426-CL. Taking P426-bts1 as an example, the *bts1* gene sequence was acquired from NCBI, and CRISPy tool [11, 50] (http://staff.biosustain.dtu.dk/laeb/crispy_cenpk/) was used to choose the specific gRNA sequence (Additional file 1: Table S5) targeting *bts1* gene in yeast BJ5464 genome. Subsequently, two primers bts1-F and bts1-R were designed, both containing gRNA sequence as homologous arms. Two fragments named bts1-1 and bts1-2 were PCR amplified with primer pairs tong-F/bts1-R and bts1-F/tong-R, respectively using P426-CL as template. Two fragments were assembled together using ClonExpress II One Step Cloning Kit and plasmid P426-bts1 containing *bts1* gRNA sequence was generated. The other four gRNA expression plasmids were obtained in the same way. These gRNA expression plasmids were transformed into yeast strain containing Cas9 expression plasmid (Addgene, USA) with 90-bp donor DNA (Additional file 1: Table S6) to construct single mutant strains.

Regarding the multiple mutants construction, triple mutation *bts1*;*rox1*;*erg9* was shown as an example. First, single mutant strain BJM-01 was cultivated in SD-galactose medium for 36 h to induce the expression of Cre recombinase. Then, yeast solution was plated in SD-glucose/5-FOA solid plate to select the positive colony that lost gRNA expression plasmid P426-bts1 containing *URA3* selection marker. After recycling of selection marker, another gRNA expression plasmid P426-rox1 and appropriate donor DNA were transformed into the above positive strain, resulting in double mutant strain BJM-14. Similarly, the *URA3* selection marker in BJM-14 was recycled through galactose induction and gRNA expression plasmid P426-erg9 was introduced into the strain, resulting in triple mutation strain BJM-24. Other multiple mutants were achieved through the reuse of 5 gRNA expression plasmids as described above (Fig. 2).

#### Overexpression of FPP biosynthetic pathway genes

To further enhance the supply of FPP, the FPP biosynthesis pathway genes (*erg10*, *erg13*, *thmg1*, *erg12*, *erg8*, *erg19*, *idi1*, *erg20*) were overexpressed in yeast genome through the plasmid P426-CL constructed in this study (Fig. 5-a). All the genes were amplified using *S. cerevisiae* genomic DNA as template. Four integration sites of *bts1*, *rox1*, *dpp1* and *lpp1* were chosen for the aforementioned 8 genes overexpression. The *ypl062w* and *yjl064w* were replaced by *dpp1* and *lpp1*, because in this study, the alterations on them did not show positive effects on valencene production as other researchers’ report [7, 14]. While knock-out of *dpp1* and *lpp1* could reduce the FPP hydrolysis and increase the substrate supplementation for valencene synthesis [37].

A bidirectional expression vector containing P_TDH3_-T_ADH1_ and P_TEF1_-T_CYC1_ was first constructed based on the plasmid YEplac181, denoted as pYM01. Genes of *erg20* and *idi1* were cloned from yeast genome. Two fragments named backone-1 and backone-2 were amplified with primer pairs cas1-F/181-bone-R and 181-bone-2-F/181-bone-2-R using pYM01 as template. These four fragments were assembled into a recombinant plasmid named pYM02 using ClonExpress MultiS One Step Cloning Kit (Vazyme, China) (Fig. 5a). The bidirectional expression fragment, T_ADH1_-ERG20-P_TDH3_-P_TEF1_-IDI1-T_CYC1_, amplified from plasmid pYM02 was transformed into strain BJM-03 as a donor DNA with gRNA expression plasmid P426-rox1. After knock-out of *rox1* gene loci and knock-in of fragment T_ADH1_-ERG20-P_TDH3_-P_TEF1_-IDI1-T_CYC1_, the overexpression of *erg20* gene and *idi1* gene was achieved. Similar method was used to obtain overexpression of other remaining genes, resulting in strain BJM-33.

#### Construction of different *CnVS* expression cassettes

To optimize the expression cassette of valencene synthase (*CnVS*), a promoter-terminator library was constructed. A series of promoters (ADH1, CYC1, HXT7, FBA1, PGK1, TDH3, and TEF1) and terminators (ADH1, CYC1, TPI1, PGI1, and FBA1) from *S. cerevisiae* genome were chosen. The fragments of promoters and terminators were amplified by PCR with primer pairs containing restriction sites. The resulting fragments were assembled into plasmid YEplac181 after digestion by different restriction enzymes, generating the promoter-terminator library. Then, valencene synthase gene was inserted between promoter and terminator using *Xba*I and *Pst*I to obtain different expression cassettes. All recombinant plasmids were transformed into wild-type strain BJ5464 to examine the valencene production.

#### Plasmid loss efficiency determination

The recyclable plasmid P426-CL containing the selection marker *URA3* was transformed into *S. cerevisiae* BJ5464. Single colony of recombinant yeast was selected and inoculated into SD-galactose medium to induce Cre recombinase expression. After incubation at 30 °C for 36 h with shaking at 200 rpm, colony-forming unit per milliliter (CFU/mL) was determined using haemocytometer through microscopic counts. Then, 0.2 mL of yeast culture with different dilution factors was plated onto SD-glucose agar plate without uracil. Cells harboring the plasmid P426-CL grew out and colony number were recorded.

The plasmid loss efficiency was calculated using the following formula:

$$ {\text{Plasmid loss efficiency}} = \frac{{A_{1} - 5DA_{2} }}{{A_{1} }} $$

*A*_1_, cell density of yeast culture, CFU/mL, *A*_2_, number of colonies per plate, *D*, dilution factor.

Triplicate measurements were performed for each set.

#### Cultivation in shake flasks

The yields of valencene-producing strains constructed in this study were evaluated in shake flask through two-phase flask cultivation. SD-glucose medium was used in the entire cultivation process. Strains were recovered from glycerol stocks by streaking on SD-glucose agar plates and pre-cultured in SD-glucose medium to exponential phase (cell density OD_600_ between 1 and 3). Two-phase flask cultivation was initiated by inoculating pre-cultured seed broth to OD_600_ = 0.05 with 10 mL SD-glucose medium in 50 mL flasks. After adding 2 mL *n*-dodecane to extract valencene, flasks were sealed with breathable microporous film. Flask cultivation was performed at 30 °C and 200 rpm.

#### Fed-batch fermentation of valencene in bioreactors

Strain BJM-45 was selected for fed-batch fermentation. Seed cultures were prepared by inoculating 250 µL of glycerol-stock into a test tube containing 5 mL SD-glucose and culturing at 30 °C for 24 h to an OD_600_ of 3–5. Then, 3–4 mL of precultures were inoculated into a 500 mL shake-flask containing 100 mL SD-glucose/and subcultured for an additional 24 h at 30 °C to an OD_600_ of 5–6. Seed cultures were transferred into a 3 L bioreactor containing 1 L SD-glucose batch medium at a 5–10% (v/v) inoculum. Valencene was extracted using 200 mL *n*-dodecane. Fermentation was carried out at 30 °C with an air flow rate of 0.8 vvm. The dissolved oxygen level was kept at 60% by adjusting the agitation speed from 250 to 500 rpm and pH was controlled at 6.0 by automatic addition of 1 M NaOH. After 36 h of fermentation when the initial glucose was fully consumed, concentrated glucose solution (400 g/L) was fed into bioreactors every 24 h to maintain the growth of strain. The fermentation was ended at 135 h when the cells were basically inactive (Additional file 1: Fig S3).

### Analytical methods

#### Biomass determination

Cell density in the medium was determined by measuring absorbance at 600 nm using an ultraviolet–visible spectrophotometer (Thermo Fisher Scientific GENESYS 10, Germany). The dry cell weight (DCW) was measured by analytical balance (Sartorius, Beijing) after the yeast solution was dried in a drying oven.

#### Extraction and analysis of valencene

##### GC-FID

The culture of valencene fermentation was covered with 20% *n*-dodecane to extract valencene secreted in the medium. To determine content of valencene, 500 μL sample collected from *n*-dodecane organic phase in the two-phase fermentation medium was mixed with 500 μL ethyl acetate and 2 μL isolongifolene. The resulting mixture was passed through a 0.22 μm sterile filter. Valencene concentration was detected by gas chromatography (GC, Agilent Technologies, 7890) equipped with a flame ionization detector (FID). An HP-5 column (30 m × 0.32 mm; 0.25 μm film thickness; Agilent Technologies) was used, with nitrogen as the carrier gas. Samples were run using the following analysis method: GC oven temperature program was maintained at 100 °C for 10 min, followed by a ramp to 200 °C at 10 °C/min, and then maintained at 200 °C for 8 min.

##### GC–MS

A GC/MS-HP7890 system (Agilent, USA) equipped with a 5975C series mass selective detector (MSD) was used to perform the GC/MS analysis. The same GC oven temperature program were used as described above for GC-FID detection. MS data were recorded at 70 eV (EI), m/z (rel. intensity in %) as TIC, total ion current. The data were collected in full scan mode (m/z 50–650). Compounds in the samples were identified by comparison of retention time and mass in the GC/MS spectra to the commercially available chemical standards and mass spectral data of the NIST Standard Reference Database.

##### Analysis of metabolites by HPLC

Glucose consumption and ethanol accumulation were determined by analyzing the glucose and ethanol concentration in the medium using high-performance liquid chromatography (HPLC) as previously described [51].

## Supplementary information

**Additional file 1:** Additional tables and figures.

## Data Availability

The datasets used or analysed during the current study are available from the corresponding author on reasonable request.
