# Supplementary material for: High production of valencene in Saccharomyces cerevisiae through metabolic engineering
Source: Microb Cell Fact. 2019 Nov 7;18:195. doi: 10.1186/s12934-019-1246-2 (PMC6839068; doi:10.1186/s12934-019-1246-2)
Supplement: Supplementary file 1 — Additional file 1: Additional tables and figures. [file 12934_2019_1246_MOESM1_ESM.docx]

# Additional files

**Table S1 Plasmids used in this study**

| Plasmid name | Description | Source |
| --- | --- | --- |
| p426 | p426-P_SNR52_-T_SUP4_, *URA3,* *Amp*^R^ | Addgene |
| P426-Cre | p426 derived, p426-P_GAL1_-Cre-T_CYC1_-P_SNR52_-T_SUP4_ | This study |
| P426-CL | p426-Cre derived, p426-loxP-Ori-loxP-P_GAL1_-Cre-T_CYC1_-P_SNR52_-T_SUP4_ | This study |
| P426-bts1 | p426-CL derived, p426-loxP-Ori-loxP-P_GAL1_-Cre-T_CYC1_-P_SNR52_-gRNA.BTS1-T_SUP4_ | This study |
| P426-rox1 | p426-CL derived, p426-loxP-Ori-loxP-P_GAL1_-Cre-T_CYC1_-P_SNR52_-gRNA.ROX1-T_SUP4_ | This study |
| P426-erg9 | p426-CL derived, p426-loxP-Ori-loxP-P_GAL1_-Cre-T_CYC1_-P_SNR52_-gRNA.ERG9-T_SUP4_ | This study |
| P426-ypl062w | p426-CL derived, p426-loxP-Ori-loxP-P_GAL1_-Cre-T_CYC1_-P_SNR52_-gRNA.YPL062W-T_SUP4_ | This study |
| P426-yjl064w | p426-CL derived, p426-loxP-Ori-loxP-P_GAL1_-Cre-T_CYC1_-P_SNR52_-gRNA.YJL064w-T_SUP4_ | This study |
| P426-dpp1 | p426-CL derived, p426-loxP-Ori-loxP-P_GAL1_-Cre-T_CYC1_-P_SNR52_-gRNA.DPP1-T_SUP4_ | This study |
| P426-lpp1 | p426-CL derived, p426-loxP-Ori-loxP-P_GAL1_-Cre-T_CYC1_-P_SNR52_-gRNA.LPP1-T_SUP4_ | This study |
| p414 | p414-CEN/ARS-P_TEF1_-Cas9-T_CYC1_, *TRP1, Amp*^R^ | Adgene |
| YEplac181 | *S. cerevisiae*-*E. coli* shuttle vector, High-copy (2μ), *LEU*2, *Amp*^R^ |  |
| pYM01 | YEplac181 derived, containing T_ADH1_-P_TDH3_-P_TEF1_-T_CYC1_ | This study |
| pYM02 | YM01 derived, containing T_ADH1_-ERG20-P_TDH3_-P_TEF1_-IDI1-T_CYC1_ | This study |
| pYM03 | YM01 derived, containing T_ADH1_-ERG8-P_TDH3_-P_TEF1_-ERG19-T_CYC1_ | This study |
| pYM04 | YM01 derived, containing T_ADH1_-ERG12-P_TDH3_-P_TEF1_-tHMG1-T_CYC1_ | This study |
| pYM05 | YM01 derived, containing T_ADH1_-ERG13-P_TDH3_-P_TEF1_-ERG10-T_CYC1_ | This study |
| pYM06 | YEplac181 derived, containing P_TDH3_-VS-T_ADH1_ | This study |
| pYM07 | YEplac181 derived, containing P_ADH1_-VS-T_ADH1_ | This study |
| pYM08 | YEplac181 derived, containing P_CYC1_-VS-T_CYC1_ | This study |
| pYM09 | YEplac181 derived, containing P_FBA1_-VS-T_CYC1_ | This study |
| pYM10 | YEplac181 derived, containing P_HXT7_-VS-T_TPI1_ | This study |
| pYM11 | YEplac181 derived, containing P_PGK1_-VS-T_ENO2_ | This study |
| pYM12 | YEplac181 derived, containing P_TDH3_-VS-T_PGI1_ | This study |
| pYM13 | YEplac181 derived, containing P_TEF1_-VS-T_FBA1_ | This study |

**Table S2 The valencene biosynthesis genes and genome editing genes used in this study**

| **Gene** | **Accession number** | **Description** | **Origin** |
| --- | --- | --- | --- |
| *CnVS* | JX040471 | valencene synthase | *Callitropsis nootkatensis* |
| *bts1* | NC_001148.4 | geranylgeranyl diphosphate synthase | *Saccharomyces cerevisiae* |
| *erg9* | NC_001140.6 | squalene synthase | *Saccharomyces cerevisiae* |
| *rox1* | NC_001148.4 | oxygen-responsive repressor | *Saccharomyces cerevisiae* |
| *ypl062w* | NC_001148.4 |  | *Saccharomyces cerevisiae* |
| *yjl064w* | NC_001142 |  | *Saccharomyces cerevisiae* |
| *dpp1* | NC_001136.10 | bifunctional diacylglycerol diphosphate phosphatase/phosphatidate phosphatase | *Saccharomyces cerevisiae* |
| *lpp1* | NC_001136.10 | phosphatidate phosphatase | *Saccharomyces cerevisiae* |
| *erg13* | NC_001145.3 | hydroxymethylglutaryl-CoA synthase | *Saccharomyces cerevisiae* |
| *erg10* | NC_001148.4 | acetyl-CoA C-acetyltransferase | *Saccharomyces cerevisiae* |
| *erg12* | NC_001145.3 | mevalonate kinase | *Saccharomyces cerevisiae* |
| *tHMG1* | NM_001182434.1 | 3-hydroxy-3-methylglutaryl-coenzyme A reductase gene | *Saccharomyces cerevisiae* |
| *erg8* | NC_001145.3 | phosphomevalonate kinase | *Saccharomyces cerevisiae* |
| *erg19* | NC_027325.1 | diphosphomevalonate decarboxylase | *Saccharomyces cerevisiae* |
| *idi1* | NC_001148.4 | isopentenyl-diphosphate delta-isomerase | *Saccharomyces cerevisiae* |
| *erg20* | NC_001142.9 | Farnesyl diphosphate synthase/dimethylallyltranstransferase | *Saccharomyces cerevisiae* |

**Table S3 *S. cerevisiae* strains used in this study**

| Strain | Parent Strain | Genotype**/**Plasmid | Source |
| --- | --- | --- | --- |
| ***E. coli*** |  |  |  |
| DH5α |  | F^-^ ϕ80 *lac*Z ΔM15 Δ(*lac*ZYA-*arg*F) U169 *rec*A1 *end*A1 *hsd*R17 (r_K_^-^, m_K_^+^) *pho*A *sup*E44 λ^-^ *thi*-1 *gyr*A96 *rel*A1 | Invitrogen |
| ***S. cerevisiae*** |  |  |  |
| BJ5464 |  | *MATα ura3-52 trp1 leu2*Δ*1 his3*Δ*200 pep4::HIS3 prb1*Δ*1.6R can1 GAL* | Invitrogen |
| BJM-00 | BJ5464 | YEplac181-P_TDH3_-VS-T_ADH1_ | This study |
| BJM-C | BJ5464 | p414-P_TEF1_-Cas9-T_CYC1_ and YEplac181-P_TDH3_-VS-T_ADH1_ | This study |
| BJM-01 | BJ5464 | *bts1::mut,* p414-P_TEF1_-Cas9-T_CYC1_ and YEplac181-P_TDH3_-VS-T_ADH1_ | This study |
| BJM-02 | BJ5464 | *rox1::mut*, p414-P_TEF1_-Cas9-T_CYC1_ and YEplac181-P_TDH3_-VS-T_ADH1_ | This study |
| BJM-03 | BJ5464 | *erg9::*Δ-220--176, p414-P_TEF1_-Cas9-T_CYC1_ and YEplac181-P_TDH3_-VS-T_ADH1_ | This study |
| BJM-04 | BJ5464 | *ypl062w::mut*, p414-P_TEF1_-Cas9-T_CYC1_ and YEplac181-P_TDH3_-VS-T_ADH1_ | This study |
| BJM-05 | BJ5464 | *yjl064w::mut*, p414-P_TEF1_-Cas9-T_CYC1_ and YEplac181-P_TDH3_-VS-T_ADH1_ | This study |
| BJM-06 | BJM-01 | *bts1::mut erg9::*Δ-220--176, p414-P_TEF1_-Cas9-T_CYC1_ and YEplac181-P_TDH3_-VS-T_ADH1_ | This study |
| BJM-07 | BJM-03 | *erg9::*Δ-220--176 *yjl064w::mut*, p414-P_TEF1_-Cas9-T_CYC1_ and YEplac181-P_TDH3_-VS-T_ADH1_ | This study |
| BJM-08 | BJM-02 | *rox1::mut yjl064w::mut*, p414-P_TEF1_-Cas9-T_CYC1_ and YEplac181-P_TDH3_-VS-T_ADH1_ | This study |
| BJM-09 | BJM-01 | *bts1::mut ypl062w::mut*, p414-P_TEF1_-Cas9-T_CYC1_ and YEplac181-P_TDH3_-VS-T_ADH1_ | This study |
| BJM-10 | BJM-01 | *bts1::mut yjl064w::mut*, p414-P_TEF1_-Cas9-T_CYC1_ and YEplac181-P_TDH3_-VS-T_ADH1_ | This study |
| BJM-11 | BJM-02 | *rox1::mut ypl062w::mut*, p414-P_TEF1_-Cas9-T_CYC1_ and YEplac181-P_TDH3_-VS-T_ADH1_ | This study |
| BJM-12 | BJM-03 | *erg9::*Δ-220--176 *ypl062w::mut*, p414-P_TEF1_-Cas9-T_CYC1_ and YEplac181-P_TDH3_-VS-T_ADH1_ | This study |
| BJM-13 | BJM-04 | *ypl062w::mut yjl064w::mut*, p414-P_TEF1_-Cas9-T_CYC1_ and YEplac181-P_TDH3_-VS-T_ADH1_ | This study |
| BJM-14 | BJM-01 | *bts1::mut rox1::mut*, p414-P_TEF1_-Cas9-T_CYC1_ and YEplac181-P_TDH3_-VS-T_ADH1_ | This study |
| BJM-15 | BJM-03 | *erg9::*Δ-220--176 *rox1::mut*, p414-P_TEF1_-Cas9-T_CYC1_ and YEplac181-P_TDH3_-VS-T_ADH1_ | This study |
| BJM-16 | BJM-14 | *bts1::mut* *rox1::mut* *yjl064w::mut*, p414-P_TEF1_-Cas9-T_CYC1_ and YEplac181-P_TDH3_-VS-T_ADH1_ | This study |
| BJM-17 | BJM-07 | *erg9::*Δ-220--176 *ypl062w::mut yjl064w::mut*, p414-P_TEF1_-Cas9-T_CYC1_ and YEplac181-P_TDH3_-VS-T_ADH1_ | This study |
| BJM-18 | BJM-11 | *rox1::mut* *ypl062w::mut yjl064w::mut*, p414-P_TEF1_-Cas9-T_CYC1_ and YEplac181-P_TDH3_-VS-T_ADH1_ | This study |
| BJM-19 | BJM-10 | *bts1::mut erg9::*Δ-220--176 *yjl064w::mut*, p414-P_TEF1_-Cas9-T_CYC1_ and YEplac181-P_TDH3_-VS-T_ADH1_ | This study |
| BJM-20 | BJM-14 | *bts1::mut* *rox1::mut ypl062w::mut*, p414-P_TEF1_-Cas9-T_CYC1_ and YEplac181-P_TDH3_-VS-T_ADH1_ | This study |
| BJM-21 | BJM-09 | *bts1::mut ypl062w::mut yjl064w::mut*, p414-P_TEF1_-Cas9-T_CYC1_ and YEplac181-P_TDH3_-VS-T_ADH1_ | This study |
| BJM-22 | BJM-07 | *erg9::*Δ-220--176 *rox1::mut yjl064w::mut*, p414-P_TEF1_-Cas9-T_CYC1_ and YEplac181-P_TDH3_-VS-T_ADH1_ | This study |
| BJM-23 | BJM-09 | *bts1::mut erg9::*Δ-220--176 *ypl062w::mut*, p414-P_TEF1_-Cas9-T_CYC1_ and YEplac181-P_TDH3_-VS-T_ADH1_ | This study |
| BJM-24 | BJM-14 | *bts1::mut erg9::*Δ-220--176 *rox1::mut*, p414-P_TEF1_-Cas9-T_CYC1_ and YEplac181-P_TDH3_-VS-T_ADH1_ | This study |
| BJM-25 | BJM-11 | *rox1::mut erg9::*Δ-220--176 *ypl062w::mut*, p414-P_TEF1_-Cas9-T_CYC1_ and YEplac181-P_TDH3_-VS-T_ADH1_ | This study |
| BJM-26 | BJM-20 | *bts1::mut erg9::*Δ-220--176 *rox1::mut ypl062w::mut*, p414-P_TEF1_-Cas9-T_CYC1_ and YEplac181-P_TDH3_-VS-T_ADH1_ | This study |
| BJM-27 | BJM-19 | *bts1::mut erg9::*Δ-220--176 *ypl062w::mut yjl064w::mut*, p414-P_TEF1_-Cas9-T_CYC1_ and YEplac181-P_TDH3_-VS-T_ADH1_ | This study |
| BJM-28 | BJM-20 | *bts1::mut rox1::mut ypl062w::mut yjl064w::mut*, p414-P_TEF1_-Cas9-T_CYC1_ and YEplac181-P_TDH3_-VS-T_ADH1_ | This study |
| BJM-29 | BJM-19 | *bts1::mut erg9::*Δ-220--176 *rox1::mut yjl064w::mut*, p414-P_TEF1_-Cas9-T_CYC1_ and YEplac181-P_TDH3_-VS-T_ADH1_ | This study |
| BJM-30 | BJM-22 | *erg9::*Δ-220--176 *rox1::mut ypl062w::mut yjl064w::mut*, p414-P_TEF1_-Cas9-T_CYC1_ and YEplac181-P_TDH3_-VS-T_ADH1_ | This study |
| BJM-31 | BJM-26 | *bts1::mut rox1::mut erg9::*Δ-220--176 *ypl062w::mut yjl064w::mut*, p414-P_TEF1_-Cas9-T_CYC1_ and YEplac181-P_TDH3_-VS-T_ADH1_ | This study |
| BJM-33 | BJM-03 | *rox1::*T_ADH1_-ERG20-P_TDH3_-P_TEF1_-IDI1*-*T_CYC1_, *bts1::*T_ADH1_-ERG8-P_TDH3_-P_TEF1_-ERG19*-*T_CYC1_, *dpp1::*T_ADH1_-ERG12-P_TDH3_-P_TEF1_-tHMG1*-*T_CYC1_, *lpp1::*T_ADH1_-ERG13-P_TDH3_-P_TEF1_-ERG10*-*T_CYC1_, *erg9::*Δ-220--176, p414-P_TEF1_-Cas9-T_CYC1_ and YEplac181-P_TDH3_-VS-T_ADH1_ | This study |
| BJM-34 | BJ5464 | YEplac181-P_ADH1_-VS-T_ADH1_ | This study |
| BJM-35 | BJ5464 | YEplac181-P_CYC1_-VS-T_CYC1_ | This study |
| BJM-36 | BJ5464 | YEplac181-P_FBA1_-VS-T_CYC1_ | This study |
| BJM-37 | BJ5464 | YEplac181-P_HXT7_-VS-T_TPI1_ | This study |
| BJM-38 | BJ5464 | YEplac181-P_PGK1_-VS-T_ENO2_ | This study |
| BJM-39 | BJ5464 | YEplac181-P_TDH3_-VS-T_PGI1_ | This study |
| BJM-40 | BJ5464 | YEplac181-P_TEF1_-VS-T_FBA1_ | This study |
| BJM-41 | BJ5464 | *erg9::*Δ-220--176, p414-P_TEF1_-Cas9-T_CYC1_ | This study |
| BJM-42 | BJM-41 | *rox1::mut erg9::*Δ-220--176, p414-P_TEF1_-Cas9-T_CYC1_ | This study |
| BJM-43 | BJM-41 | *rox1::*T_ADH1_-ERG20-P_TDH3_-P_TEF1_-IDI1*-*T_CYC1_, *bts1::*T_ADH1_-ERG8-P_TDH3_-P_TEF1_-ERG19*-*T_CYC1_, *dpp1::*T_ADH1_-ERG12-P_TDH3_-P_TEF1_-tHMG1*-*T_CYC1_, *lpp1::*T_ADH1_-ERG13-P_TDH3_-P_TEF1_-ERG10*-*T_CYC1_, *erg9::*Δ-220--176, p414-P_TEF1_-Cas9-T_CYC1_ | This study |
| BJM-44 | BJM-42 | *rox1::mut erg9::*Δ-220--176, p414-P_TEF1_-Cas9-T_CYC1_ and YEplac181-P_HXT7_-VS-T_TPI1_ | This study |
| BJM-45 | BJM-43 | *rox1::*T_ADH1_-ERG20-P_TDH3_-P_TEF1_-IDI1*-*T_CYC1_, *bts1::*T_ADH1_-ERG8-P_TDH3_-P_TEF1_-ERG19*-*T_CYC1_, *dpp1::*T_ADH1_-ERG12-P_TDH3_-P_TEF1_-tHMG1*-*T_CYC1_, *lpp1::*T_ADH1_-ERG13-P_TDH3_-P_TEF1_-ERG10*-*T_CYC1_, *erg9::*Δ-220--176, p414-P_TEF1_-Cas9-T_CYC1_ and YEplac181-P_HXT7_-VS-T_TPI1_ | This study |

**Table S4 Primers used in this study**

| Primers | Sequence (5′-3′) | Description |
| --- | --- | --- |
| tong-F | TAATAATGGTTTCTTAGTATGA | Construction of gRNA expression plasmid |
| tong-R | ACTAAGAAACCATTATTATCAT |  |
| bts1-F | TGATCAATAATGATCCTGTTGTTTTAGAGCTAGAAATA | Construction of gRNA expression plasmid P426-bts1 |
| bts1-R | AACAGGATCATTATTGATCAGATCATTTATCTTTCACTGC |  |
| rox1-F | ACAGGATCTTAATAGACGAAGTTTTAGAGCTAGAAATA | Construction of gRNA expression plasmid P426-rox1 |
| rox1-R | TTCGTCTATTAAGATCCTGTGATCATTTATCTTTCACTG |  |
| erg9-F | TTTTCCACTGCACTTTGCATGTTTTAGAGCTAGAAATAGCA | Construction of gRNA expression plasmid P426-erg9 |
| erg9-R | ATGCAAAGTGCAGTGGAAAAGATCATTTATCTTTCACTG |  |
| ypl062w-F | GCACGTCGCCGTGGCTGATGGTTTTAGAGCTAGAAATA | Construction of gRNA expression plasmid P426-ypl062w |
| ypl062w-R | CATCAGCCACGGCGACGTGCGATCATTTATCTTTCACTGC |  |
| yjl064w-F | ACGACAGCGTGAGTTCATCTGTTTTAGAGCTAGAAATA | Construction of gRNA expression plasmid P426-yjl064w |
| yjl064w-R | AGATGAACTCACGCTGTCGTGATCATTTATCTTTCACTGC |  |
| bts1-M-F | AAAATCAATGGAGGCCAAGATAGATGAGCTGATCAATAATGATCCTGTTTAACCAGCCA | *bts1* donor DNA cloning |
| bts1-M-R | ATTATAAGGTTTTGAAATCAAGCTTTCATTTTGGCTGGTTAAACAGGATCATTATTGAT |  |
| rox1- M-F | GCATTTATTCTGTTCAGACAGCACTACCACAGGATCTTAATAGACGAATAACCGCTCAA | *rox1* donor DNA cloning |
| rox1- M-R | AAATGTTTGAATTATGGGGTATTTCCACACCTTGAGCGGTTATTCGTCTATTAAGATCC |  |
| UAS-M-F | CTCTGACTCAGTACATTTCATAGCCCATCTTCAACAACAATACCGACTTATCGGAAGGC | *erg9* donor DNA cloning |
| UAS-M-R | GCTCGTTTAGGCACTAAACCCAAAACCGATAACGCCTTCCGATAAGTCGGTATTGTT |  |
| ypl062w- M-F | GGTGAAGATACCATTGTAGAAGCAACCAGCACGTCGCCGTGGCTGATGTAACTCCTCTT | *ypl062w* donor DNA cloning |
| ypl062w- M-R | GGCCACTGCCCCTCTTTTCTGCGGCCCGGGCAAGAGGAGTTACATCAGCCACGGCGACG |  |
| yjl064w-M-F | TGCACAATTGCGGCAGAGATGTCATCGTACGACAGCGTGAGTTCATCTTAAGCGGCGGT | *yjl064w* donor DNA cloning |
| yjl064w-M-R | ATAGGCAACAGCAGCAGCAACAACAACAGGTACCGCCGCTTAAGATGAACTCACGCTGT |  |
| PB-F | TCGTATAATGTATGCTATACGAAGTTATAATGTGCGCGGAACCCCTAT | 3’ loxP direct repeat cloning |
| Ori-R | GTATAGCATACATTATACGAAGTTATTCCCCGAAAAGTGCCACCTGA |  |
| PB-R | TCGTATAGCATACATTATACGAAGTTATACTTATATGCGTCTATTTATGTAG | 5’ loxP direct repeat cloning |
| Ori-F | GTATAATGTATGCTATACGAAGTTATACGCATTTAAGCATAAACAC |  |
| Cre-F | GTTACCGAGCTCGTTACCGGATCCATGAGTAACCTGCTGACAGTG | *Cre* gene cloning |
| Cre-R | GAGCGATCTAGAGAGCGACCCGGGTTAATCCCCATCTTCTAATAACC |  |
| GAL1-F | CTTTAATTTGCGGCCGGTACCAGTACGGATTAGAAGCCGC | P_GAL1_-Cre-T_CYC1_ fragment cloning |
| CYC1-R | CTATAGGGCGAATTGGGTACCGCAAATTAAAGCCTTCGAG |  |
| VS-F | GTTACCGAGCTCGTTACCGGATCCATGGCTGAAATGTTCAACGGA | *CnVS* gene cloning |
| VS-R | GAGCGATCTAGAGAGCGACCCGGGTTAGGGAATAATCGGTTCGAC |  |
| cas1-F | TCTCTTCTCGAGTCATGTAATTAG | Backbone-1 fragment cloning |
| 181-bone-R | CCCGGGTCGCTCTCTAGAAGCT |  |
| 181-bone-2-F | ATCCGGTAACGAGCTCTTTGTTTGTTTATGTG | Backbone-2 fragment cloning |
| 181-bone-2-R | ACTAGTTCTAGAAAACTTAGATTAG |  |
| ADH1-ERG20-F | AAGCTTCTAGAGAGCGACCCGGGCTATTTGCTTCTCTTGTAAA | *erg20* gene cloning |
| TDH3-ERG20-R | ACAAAGAGCTCGTTACCGGATCCATGGCTTCAGAAAAAGAA |  |
| TEF1-IDI1-F | ATCTAAGTTTTCTAGAACTAGTATGACTGCCGACAACAATAG | *idi1* gene cloning |
| CYC1-IDI1-R | ATTACATGACTCGAGAAGAGATTATAGCATTCTATGAATTT |  |
| ADH1-ERG8-F | AAGCTTCTAGAGAGCGACCCGGGTTATTTATCAAGATAAGTTTCCG | *erg8* gene cloning |
| TDH3-ERG8-R | ACAAAGAGCTCGTTACCGGATCCATGTCAGAGTTGAGAGCC |  |
| TEF1-ERG19-F | ATCTAAGTTTTCTAGAACTAGTATGACCGTTTACACAGCA | *erg19* gene cloning |
| CYC1-ERG19-R | ATTACATGACTCGAGAAGAGATTATTCCTTTGGTAGACC |  |
| ADH1-ERG12-F | AAGCTTCTAGAGAGCGACCCGGGTTATGAAGTCCATGGTAA | *erg12* gene cloning |
| TDH3-ERG12-R | ACAAAGAGCTCGTTACCGGATCCATGTCATTACCGTTCTTA |  |
| TEF1-tHMG1-F | ATCTAAGTTTTCTAGAACTAGTATGGACCAATTGGTGAAA | *thmg1* gene cloning |
| CYC1-tHMG1-R | ATTACATGACTCGAGAAGAGATTAGGATTTAATGCAGGT |  |
| ADH1-ERG13-F | AAGCTTCTAGAGAGCGACCCGGGTTATTTTTTAACATCGTAAGATC | *erg13* gene cloning |
| TDH3-ERG13-R | ACAAAGAGCTCGTTACCGGATCCATGAAACTCTCAACTAAA |  |
| TEF1-ERG10-F | ATCTAAGTTTTCTAGAACTAGTATGTCTCAGAACGTTTAC | *erg10* gene cloning |
| CYC1-ERG10-F | ATTACATGACTCGAGAAGAGATCATATCTTTTCAATGACAA |  |
| rox1-ADH1-F | AGACCCAAGAACGCATTTATTCTGTTCAGACAGCACTACCCATAGGGTAGGGGAATTT | T_ADH1_-ERG20-P_TDH3_-P_TEF1_-IDI1-T_CYC1_ fragment cloning |
| rox1-CYC1-R | TGGACCGCTCAAGGTGTGGAAATACCCCATAATTCAAACAGCAAATTAAAGCCTTCGA |  |
| bts1-ADH1-F | TACGAGTCTGGAAAATCAATGGAGGCCAAGATAGATGAGCCATAGGGTAGGGGAATTT | T_ADH1_-ERG8-P_TDH3_-P_TEF1_-ERG19-T_CYC1_ fragment cloning |
| bts1-CYC1-R | TATAAGGTTTTGAAATCAAGCTTTCATTTTGGCTGGACCAGCAAATTAAAGCCTTCGA |  |
| dpp1-ADH1-F | CAAACGACCAAAATGAACAGAGTTTCGTTTATTAAAACGCCATAGGGTAGGGGAATTT | T_ADH1_-ERG12-P_TDH3_-P_TEF1_-tHMG1-T_CYC1_ fragment cloning |
| dpp1-CYC1-R | GTATCATGATAATGAGCAAAAAGACATCTTCTAATCTCCAGCAAATTAAAGCCTTCGA |  |
| lpp1-ADH1-F | CATGATAATTGGTCTATGTACGATATTATTCCTCTATTCGCATAGGGTAGGGGAATTT | T_ADH1_-ERG13-P_TDH3_-P_TEF1_-ERG10-T_CYC1_ fragment cloning |
| lpp1-CYC1-R | TATACTGGGGTCATCAAGACTAAATTCGATGTTTTGGCCCGCAAATTAAAGCCTTCGA |  |
| ADH1-P-F | TTACAGGATCCCAACTTCTTTTCTTTTTTTTTCTTTTCTCTCTCC | ADH1 promoter cloning |
| ADH1-P-R | GAGCGATCTAGAAGTTGATTGTATGCTTGGTATAG |  |
| CYC1-P-F | TTACAGGATCCTTGGAAAACCAAGAAATGAAT | CYC1 promoter cloning |
| CYC1-P-R | GAGCGATCTAGATATTAATTTAGTGTGTGTATTTG |  |
| HXT7-P-F | TTACAGGATCCCTCGTAGGAAAAATTTCGG | HXT7 promoter cloning |
| HXT7-P-R | GAGCGATCTAGATTTTTGATTAAAATTAAAAAAACTTTTTG |  |
| FBA1-P-F | TTACAGGATCCATAACAATACTGACAGTACTA | FBA1 promoter cloning |
| FBA1-P-R | GAGCGATCTAGATTTGAATATGTATTACTTGGTTAT |  |
| PGK1-P-F | TTACAGGATCCTATTTTAGATTCCTGACTTCAAC | PGK1 promoter cloning |
| PGK1-P-R | GAGCGATCTAGATGTTTTTATATTTGTTGTA |  |
| TDH3-P-F | TTACAGGATCCACAGTTTATTCCTGGCATCCAC | TDH3 promoter cloning |
| TDH3-P-R | GAGCGATCTAGATTTGTTTGTTTATGTGTGTT |  |
| TEF1-P-F | TTACAGGATCCCATAGCTTCAAAATGTTTCTAC | TEF1 promoter cloning |
| TEF1-P-R | GAGCGATCTAGACTTAGATTAGATTGCTATGCTTTC |  |
| ADH1-T-F | TTTACACTGCAGAGCTTTGGACTTCTTCGCC | ADH1 terminator cloning |
| ADH1-T-R | GAGCGAAAGCTTCATAGGGTAGGGGAATTTCGAC |  |
| CYC1-T-F | TTTACACTGCAGGGGCCGCATCATGTAATTAG | CYC1 terminator cloning |
| CYC1-T-R | GAGCGAAAGCTTGCAAATTAAAGCCTTCGAG |  |
| TPI1-T-F | TTTACACTGCAGGATTAATATAATTATATAAAAATATTATC | TPI1 terminator cloning |
| TPI1-T-R | GAGCGAAAGCTTCTATATAACAGTTGAAATTTGG |  |
| PGI1-T-F | TTTACACTGCAGAACAAATCGCTCTTAAATATATACC | PGI1 terminator cloning |
| PGI1-T-R | GAGCGAAAGCTTGGTATACTGGAGGCTTCATGAGTTATG |  |
| FBA1-T-F | TTTACACTGCAGAGTTAATTCAAATTAATTGATATAG | FBA1 terminator cloning |
| FBA1-T-R | GAGCGAAAGCTTAGTAAGCTACTATGAAAGACTTTAC |  |

Homologous arms are underlined.

**Table S5 gRNA sequence and off-targets in yeast BJ5464 genome**

| **Targeted Gene** | **gRNA sequence (5′-3′)** | **Exact match** | **1 bp mismatch** | **2 bp mismatch** |
| --- | --- | --- | --- | --- |
| *bts1* | TGATCAATAATGATCCTGTT | 1 | 1 | 19 |
| *rox1* | ACAGGATCTTAATAGACGAA | 1 | 1 | 7 |
| *erg9* | TTTTCCACTGCACTTTGCAT | 1 | 1 | 3 |
| *ypl062w* | GCACGTCGCCGTGGCTGATG | 1 | 1 | 12 |
| *yjl064w* | ACGACAGCGTGAGTTCATCT | 1 | 1 | 18 |
| *dpp1* | CTTTCAACATAGGGGCGAAA | 1 | 1 | 5 |
| *lpp1* | GAGATATCCCTGGTACCTAG | 1 | 1 | 0 |

## Table S6 The donor DNA used for homologous recombination repair of knock-out genes

| **Targeted Gene** | **Sequence (5′-3′)** |
| --- | --- |
| *bts1* | AAAATCAATGGAGGCCAAGATAGATGAGCTGATCAATAATGATCCTGTTTAACCAGCCAAAATGAAAGCTTGATTTCAAAACCTTATAAT |
| *rox1* | GCATTTATTCTGTTCAGACAGCACTACCACAGGATCTTAATAGACGAATAACCGCTCAAGGTGTGGAAATACCCCATAATTCAAACATTT |
| *erg9* | CTCTGACTCAGTACATTTCATAGCCCATCTTCAACAACAATACCGACTTATCGGAAGGCGTTATCGGTTTTGGGTTTAGTGCCTAAACGAGC |
| *ypl062w* | GGTGAAGATACCATTGTAGAAGCAACCAGCACGTCGCCGTGGCTGATGTAACTCCTCTTGCCCGGGCCGCAGAAAAGAGGGGCAGTGGCC |
| *yjl064w* | TGCACAATTGCGGCAGAGATGTCATCGTACGACAGCGTGAGTTCATCTTAAGCGGCGGTACCTGTTGTTGTTGCTGCTGCTGTTGCCTAT |


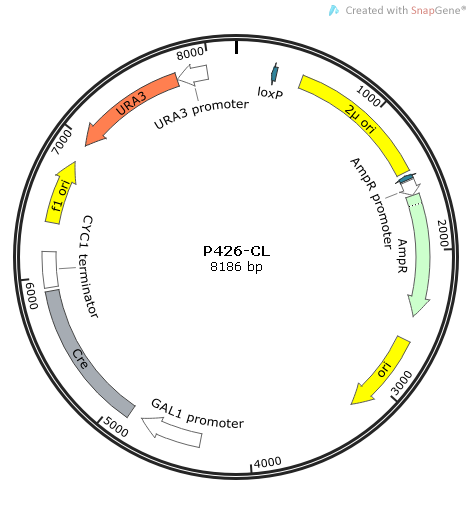


P426-CL

8186 bp

## Fig S1 Plasmid map of P426-CL.


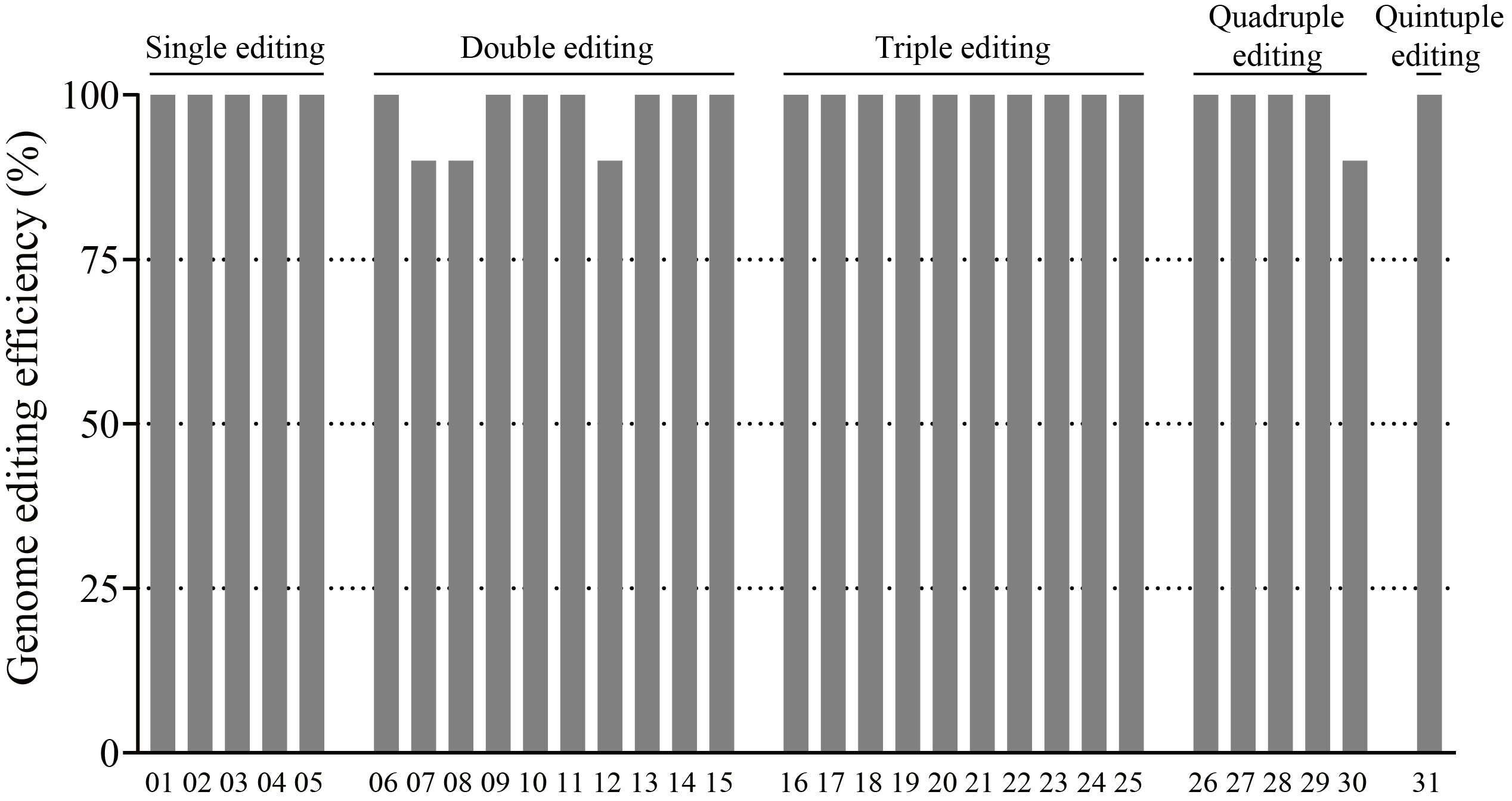


## Fig S2 Genome editing efficiencies mediated by plasmid P426-CL

Total of 31 genome editing strains were acquired using CRISPR/Cas9 system mediated by recyclable gRNA expression plasmid. To evaluate the efficiency of recyclable CRISPR/Cas9 system, 10 colonies of each genome editing strain were picked for colony PCR and sequencing. Strains from BJM-01 to BJM-31 were abbreviated from 01 to 31.


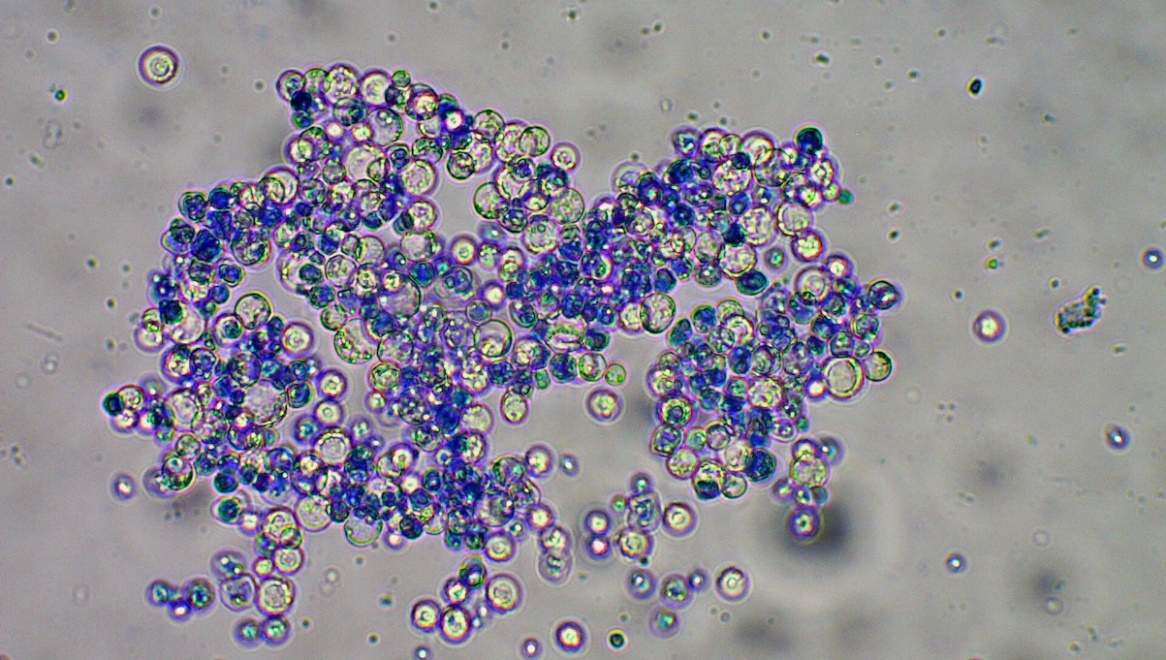


## Fig S3 The microscopy result of the yeast cells dyed with methylene blue at the later period of fed-batch fermentation.

The yeast cells at the later period of fed-batch fermentation were collected and stained with 0.2 % methylene blue (mixture of 30 mL 1% methylene blue ethanol solution and 100 mL 0.01% KOH aqueous solution). Microscopic observations were performed using a BA210 light microscope (Motic, Xiamen, China) at 400× magnification.

## Sequence of P426-CL

GACGAAAGGGCCTCGTGATACGCCTATTTTTATAGGTTAATGTCATGATAATAATGGTTTCTTAGTATGATCCAATATCAAAGGAAATGATAGCATTGAAGGATGAGACTAATCCAATTGAGGAGTGGCAGCATATAGAACAGCTAAAGGGTAGTGCTGAAGGAAGCATACGATACCCCGCATGGAATGGGATAATATCACAGGAGGTACTAGACTACCTTTCATCCTACATAAATAGACGCATATAAGTATAACTTCGTATAATGTATGCTATACGAAGTTATACGCATTTAAGCATAAACACGCACTATGCCGTTCTTCTCATGTATATATATATACAGGCAACACGCAGATATAGGTGCGACGTGAACAGTGAGCTGTATGTGCGCAGCTCGCGTTGCATTTTCGGAAGCGCTCGTTTTCGGAAACGCTTTGAAGTTCCTATTCCGAAGTTCCTATTCTCTAGAAAGTATAGGAACTTCAGAGCGCTTTTGAAAACCAAAAGCGCTCTGAAGACGCACTTTCAAAAAACCAAAAACGCACCGGACTGTAACGAGCTACTAAAATATTGCGAATACCGCTTCCACAAACATTGCTCAAAAGTATCTCTTTGCTATATATCTCTGTGCTATATCCCTATATAACCTACCCATCCACCTTTCGCTCCTTGAACTTGCATCTAAACTCGACCTCTACATTTTTTATGTTTATCTCTAGTATTACTCTTTAGACAAAAAAATTGTAGTAAGAACTATTCATAGAGTGAATCGAAAACAATACGAAAATGTAAACATTTCCTATACGTAGTATATAGAGACAAAATAGAAGAAACCGTTCATAATTTTCTGACCAATGAAGAATCATCAACGCTATCACTTTCTGTTCACAAAGTATGCGCAATCCACATCGGTATAGAATATAATCGGGGATGCCTTTATCTTGAAAAAATGCACCCGCAGCTTCGCTAGTAATCAGTAAACGCGGGAAGTGGAGTCAGGCTTTTTTTATGGAAGAGAAAATAGACACCAAAGTAGCCTTCTTCTAACCTTAACGGACCTACAGTGCAAAAAGTTATCAAGAGACTGCATTATAGAGCGCACAAAGGAGAAAAAAAGTAATCTAAGATGCTTTGTTAGAAAAATAGCGCTCTCGGGATGCATTTTTGTAGAACAAAAAAGAAGTATAGATTCTTTGTTGGTAAAATAGCGCTCTCGCGTTGCATTTCTGTTCTGTAAAAATGCAGCTAGATTCTTTGTTTGAAAAATTAGCGCTCTCGCGTTGCATTTTTGTTTTACAAAAATGAAGCACAGATTCTTCGTTGGTAAAATAGCGCTTTCGCGTTGCATTTCTGTTCTGTAAAAATGCAGCTCAGATTCTTTGTTTGAAAAATTAGCGCTCTCGCGTTGCATTTTTGTTCTACAAAATGAAGCACAGATGCTTCGTTCAGGTGGCACTTTTCGGGGAATAACTTCGTATAATGTATGCTATACGAAGTTATAATGTGCGCGGAACCCCTATTTGTTTATTTTTCTAAATACATTCAAATATGTATCCGCTCATGAGACAATAACCCTGATAAATGCTTCAATAATATTGAAAAAGGAAGAGTATGAGTATTCAACATTTCCGTGTCGCCCTTATTCCCTTTTTTGCGGCATTTTGCCTTCCTGTTTTTGCTCACCCAGAAACGCTGGTGAAAGTAAAAGATGCTGAAGATCAGTTGGGTGCACGAGTGGGTTACATCGAACTGGATCTCAACAGCGGTAAGATCCTTGAGAGTTTTCGCCCCGAAGAACGTTTTCCAATGATGAGCACTTTTAAAGTTCTGCTATGTGGCGCGGTATTATCCCGTATTGACGCCGGGCAAGAGCAACTCGGTCGCCGCATACACTATTCTCAGAATGACTTGGTTGAGTACTCACCAGTCACAGAAAAGCATCTTACGGATGGCATGACAGTAAGAGAATTATGCAGTGCTGCCATAACCATGAGTGATAACACTGCGGCCAACTTACTTCTGACAACGATCGGAGGACCGAAGGAGCTAACCGCTTTTTTGCACAACATGGGGGATCATGTAACTCGCCTTGATCGTTGGGAACCGGAGCTGAATGAAGCCATACCAAACGACGAGCGTGACACCACGATGCCTGTAGCAATGGCAACAACGTTGCGCAAACTATTAACTGGCGAACTACTTACTCTAGCTTCCCGGCAACAATTAATAGACTGGATGGAGGCGGATAAAGTTGCAGGACCACTTCTGCGCTCGGCCCTTCCGGCTGGCTGGTTTATTGCTGATAAATCTGGAGCCGGTGAGCGTGGGTCTCGCGGTATCATTGCAGCACTGGGGCCAGATGGTAAGCCCTCCCGTATCGTAGTTATCTACACGACGGGGAGTCAGGCAACTATGGATGAACGAAATAGACAGATCGCTGAGATAGGTGCCTCACTGATTAAGCATTGGTAACTGTCAGACCAAGTTTACTCATATATACTTTAGATTGATTTAAAACTTCATTTTTAATTTAAAAGGATCTAGGTGAAGATCCTTTTTGATAATCTCATGACCAAAATCCCTTAACGTGAGTTTTCGTTCCACTGAGCGTCAGACCCCGTAGAAAAGATCAAAGGATCTTCTTGAGATCCTTTTTTTCTGCGCGTAATCTGCTGCTTGCAAACAAAAAAACCACCGCTACCAGCGGTGGTTTGTTTGCCGGATCAAGAGCTACCAACTCTTTTTCCGAAGGTAACTGGCTTCAGCAGAGCGCAGATACCAAATACTGTCCTTCTAGTGTAGCCGTAGTTAGGCCACCACTTCAAGAACTCTGTAGCACCGCCTACATACCTCGCTCTGCTAATCCTGTTACCAGTGGCTGCTGCCAGTGGCGATAAGTCGTGTCTTACCGGGTTGGACTCAAGACGATAGTTACCGGATAAGGCGCAGCGGTCGGGCTGAACGGGGGGTTCGTGCACACAGCCCAGCTTGGAGCGAACGACCTACACCGAACTGAGATACCTACAGCGTGAGCTATGAGAAAGCGCCACGCTTCCCGAAGGGAGAAAGGCGGACAGGTATCCGGTAAGCGGCAGGGTCGGAACAGGAGAGCGCACGAGGGAGCTTCCAGGGGGAAACGCCTGGTATCTTTATAGTCCTGTCGGGTTTCGCCACCTCTGACTTGAGCGTCGATTTTTGTGATGCTCGTCAGGGGGGCGGAGCCTATGGAAAAACGCCAGCAACGCGGCCTTTTTACGGTTCCTGGCCTTTTGCTGGCCTTTTGCTCACATGTTCTTTCCTGCGTTATCCCCTGATTCTGTGGATAACCGTATTACCGCCTTTGAGTGAGCTGATACCGCTCGCCGCAGCCGAACGACCGAGCGCAGCGAGTCAGTGAGCGAGGAAGCGGAAGAGCGCCCAATACGCAAACCGCCTCTCCCCGCGCGTTGGCCGATTCATTAATGCAGCTGGCACGACAGGTTTCCCGACTGGAAAGCGGGCAGTGAGCGCAACGCAATTAATGTGAGTTACCTCACTCATTAGGCACCCCAGGCTTTACACTTTATGCTTCCGGCTCCTATGTTGTGTGGAATTGTGAGCGGATAACAATTTCACACAGGAAACAGCTATGACCATGATTACGCCAAGCGCGCAATTAACCCTCACTAAAGGGAACAAAAGCTGGAGCTTCTTTGAAAAGATAATGTATGATTATGCTTTCACTCATATTTATACAGAAACTTGATGTTTTCTTTCGAGTATATACAAGGTGATTACATGTACGTTTGAAGTACAACTCTAGATTTTGTAGTGCCCTCTTGGGCTAGCGGTAAAGGTGCGCATTTTTTCACACCCTACAATGTTCTGTTCAAAAGATTTTGGTCAAACGCTGTAGAAGTGAAAGTTGGTGCGCATGTTTCGGCGTTCGAAACTTCTCCGCAGTGAAAGATAAATGATCTGATCAATAATGATCCTGTTGTTTTAGAGCTAGAAATAGCAAGTTAAAATAAGGCTAGTCCGTTATCAACTTGAAAAAGTGGCACCGAGTCGGTGGTGCTTTTTTTGTTTTTTATGTCTTCGAGTCATGTAATTAGTTATGTCACGCTTACGTTCACGCCCTCCCCCCACATCCGCTCTAACCGAAAAGGAAGGAGTTAGACAACCTGAAGTCTAGGTCCCTATTTATTTTTTTATAGTTATGTTAGTATTAAGAACGTTATTTATATTTCAAATTTTTCTTTTTTTTCTGTACAGACGCGTGTACGCATGTAACATTATACTGAAAACCTTGCTTGAGAAGGTTTTGGGACGCTCGAAGGCTTTAATTTGCGGCCGGTACCAGTACGGATTAGAAGCCGCCGAGCGGGTGACAGCCCTCCGAAGGAAGACTCTCCTCCGTGCGTCCTCGTCTTCACCGGTCGCGTTCCTGAAACGCAGATGTGCCTCGCGCCGCACTGCTCCGAACAATAAAGATTCTACAATACTAGCTTTTATGGTTATGAAGAGGAAAAATTGGCAGTAACCTGGCCCCACAAACCTTCAAATGAACGAATCAAATTAACAACCATAGGATGATAATGCGATTAGTTTTTTAGCCTTATTTCTGGGGTAATTAATCAGCGAAGCGATGATTTTTGATCTATTAACAGATATATAAATGCAAAAACTGCATAACCACTTTAACTAATACTTTCAACATTTTCGGTTTGTATTACTTCTTATTCAAATGTAATAAAAGTATCAACAAAAAATTGTTAATATACCTCTATACTTTAACGTCAAGGAGAAAAAACCCCGGATCGGACTACTAGCAGCTGTAATACGACTCACTATAGGGAATATTAAGCTTGGTACCGAGCTCGTTACCGGATCCATGAGTAACCTGCTGACAGTGCATCAGAACTTGCCTGCATTACCTGTTGATGCAACAAGTGATGAAGTGAGAAAGAACCTGATGGATATGTTCAGAGACAGACAGGCATTTTCGGAGCATACATGGAAGATGCTTCTGAGTGTTTGTAGAAGCTGGGCTGCATGGTGTAAATTGAACAACAGAAAGTGGTTCCCAGCAGAACCAGAAGATGTTAGAGATTACTTGCTATATCTTCAGGCAAGAGGTCTAGCAGTGAAGACTATCCAGCAACATTTGGGACAACTAAACATGCTTCACAGAAGGAGTGGATTGCCTAGACCATCAGATTCAAATGCTGTGAGCCTTGTAATGAGAAGAATCAGGAAAGAGAACGTGGACGCAGGTGAAAGAGCTAAACAAGCACTAGCTTTTGAGAGAACGGATTTCGACCAAGTTAGATCACTTATGGAGAATTCCGACAGATGTCAAGACATCAGAAATTTGGCGTTTTTGGGGATTGCGTATAATACCTTGCTACGTATCGCCGAAATTGCCAGGATAAGGGTTAAAGACATTTCCCGTACGGACGGTGGAAGAATGTTGATACACATTGGCAGAACTAAGACCTTGGTCTCGACAGCAGGTGTTGAGAAAGCTTTATCTTTGGGTGTAACCAAATTAGTCGAAAGGTGGATTTCCGTAAGCGGTGTAGCTGATGATCCAAATAATTACTTATTTTGCCGTGTCCGTAAAAATGGTGTCGCCGCTCCGTCTGCTACTTCTCAATTATCCACCAGAGCTTTAGAAGGTATTTTTGAAGCGACTCACAGGTTAATATATGGCGCGAAAGACGATTCTGGTCAAAGGTACTTAGCCTGGTCTGGACATTCTGCTCGTGTAGGAGCTGCTAGGGATATGGCTCGTGCCGGGGTTTCAATACCCGAAATAATGCAAGCCGGTGGCTGGACTAATGTTAATATTGTTATGAATTATATTCGTACTTTAGATTCAGAAACAGGCGCCATGGTCAGGTTATTAGAAGATGGGGATTAACCCGGGTCGCTCTCTAGAGGGCCGCATCATGTAATTAGTTATGTCACGCTTACATTCACGCCCTCCCCCCACATCCGCTCTAACCGAAAAGGAAGGAGTTAGACAACCTGAAGTCTAGGTCCCTATTTATTTTTTTATAGTTATGTTAGTATTAAGAACGTTATTTATATTTCAAATTTTTCTTTTTTTTCTGTACAGACGCGTGTACGCATGTAACATTATACTGAAAACCTTGCTTGAGAAGGTTTTGGGACGCTCGAAGGCTTTAATTTGCGGTACCCAATTCGCCCTATAGTGAGTCGTATTACGCGCGCTCACTGGCCGTCGTTTTACAACGTCGTGACTGGGAAAACCCTGGCGTTACCCAACTTAATCGCCTTGCAGCACATCCCCCTTTCGCCAGCTGGCGTAATAGCGAAGAGGCCCGCACCGATCGCCCTTCCCAACAGTTGCGCAGCCTGAATGGCGAATGGCGCGACGCGCCCTGTAGCGGCGCATTAAGCGCGGCGGGTGTGGTGGTTACGCGCAGCGTGACCGCTACACTTGCCAGCGCCCTAGCGCCCGCTCCTTTCGCTTTCTTCCCTTCCTTTCTCGCCACGTTCGCCGGCTTTCCCCGTCAAGCTCTAAATCGGGGGCTCCCTTTAGGGTTCCGATTTAGTGCTTTACGGCACCTCGACCCCAAAAAACTTGATTAGGGTGATGGTTCACGTAGTGGGCCATCGCCCTGATAGACGGTTTTTCGCCCTTTGACGTTGGAGTCCACGTTCTTTAATAGTGGACTCTTGTTCCAAACTGGAACAACACTCAACCCTATCTCGGTCTATTCTTTTGATTTATAAGGGATTTTGCCGATTTCGGCCTATTGGTTAAAAAATGAGCTGATTTAACAAAAATTTAACGCGAATTTTAACAAAATATTAACGTTTACAATTTCCTGATGCGGTATTTTCTCCTTACGCATCTGTGCGGTATTTCACACCGCATAGGGTAATAACTGATATAATTAAATTGAAGCTCTAATTTGTGAGTTTAGTATACATGCATTTACTTATAATACAGTTTTTTAGTTTTGCTGGCCGCATCTTCTCAAATATGCTTCCCAGCCTGCTTTTCTGTAACGTTCACCCTCTACCTTAGCATCCCTTCCCTTTGCAAATAGTCCTCTTCCAACAATAATAATGTCAGATCCTGTAGAGACCACATCATCCACGGTTCTATACTGTTGACCCAATGCGTCTCCCTTGTCATCTAAACCCACACCGGGTGTCATAATCAACCAATCGTAACCTTCATCTCTTCCACCCATGTCTCTTTGAGCAATAAAGCCGATAACAAAATCTTTGTCGCTCTTCGCAATGTCAACAGTACCCTTAGTATATTCTCCAGTAGATAGGGAGCCCTTGCATGACAATTCTGCTAACATCAAAAGGCCTCTAGGTTCCTTTGTTACTTCTTCTGCCGCCTGCTTCAAACCGCTAACAATACCTGGGCCCACCACACCGTGTGCATTCGTAATGTCTGCCCATTCTGCTATTCTGTATACACCCGCAGAGTACTGCAATTTGACTGTATTACCAATGTCAGCAAATTTTCTGTCTTCGAAGAGTAAAAAATTGTACTTGGCGGATAATGCCTTTAGCGGCTTAACTGTGCCCTCCATGGAAAAATCAGTCAAGATATCCACATGTGTTTTTAGTAAACAAATTTTGGGACCTAATGCTTCAACTAACTCCAGTAATTCCTTGGTGGTACGAACATCCAATGAAGCACACAAGTTTGTTTGCTTTTCGTGCATGATATTAAATAGCTTGGCAGCAACAGGACTAGGATGAGTAGCAGCACGTTCCTTATATGTAGCTTTCGACATGATTTATCTTCGTTTCCTGCAGGTTTTTGTTCTGTGCAGTTGGGTTAAGAATACTGGGCAATTTCATGTTTCTTCAACACTACATATGCGTATATATACCAATCTAAGTCTGTGCTCCTTCCTTCGTTCTTCCTTCTGTTCGGAGATTACCGAATCAAAAAAATTTCAAGGAAACCGAAATCAAAAAAAAGAATAAAAAAAAAATGATGAATTGAATTGAAAAGCTGTGGTATGGTGCACTCTCAGTACAATCTGCTCTGATGCCGCATAGTTAAGCCAGCCCCGACACCCGCCAACACCCGCTGACGCGCCCTGACGGGCTTGTCTGCTCCCGGCATCCGCTTACAGACAAGCTGTGACCGTCTCCGGGAGCTGCATGTGTCAGAGGTTTTCACCGTCATCACCGAAACGCGCGA
